# Dual role of starvation signaling in promoting growth and recovery

**DOI:** 10.1371/journal.pbio.2002039

**Published:** 2017-12-13

**Authors:** Yonat Gurvich, Dena Leshkowitz, Naama Barkai

**Affiliations:** 1 Department of Molecular Genetics, Weizmann Institute of Science, Rehovot, Israel; 2 Life Sciences Core Facilities, Weizmann Institute of Science, Rehovot, Israel; Massachusetts Institute of Technology, United States of America

## Abstract

Growing cells are subject to cycles of nutrient depletion and repletion. A shortage of nutrients activates a starvation program that promotes growth in limiting conditions. To examine whether nutrient-deprived cells prepare also for their subsequent recovery, we followed the transcription program activated in budding yeast transferred to low-phosphate media and defined its contribution to cell growth during phosphate limitation and upon recovery. An initial transcription wave was induced by moderate phosphate depletion that did not affect cell growth. A second transcription wave followed when phosphate became growth limiting. The starvation program contributed to growth only in the second, growth-limiting phase. Notably, the early response, activated at moderate depletion, promoted recovery from starvation by increasing phosphate influx upon transfer to rich medium. Our results suggest that cells subject to nutrient depletion prepare not only for growth in the limiting conditions but also for their predicted recovery once nutrients are replenished.

## Author summary

Cells must have sufficient nutrients in order to perform their different functions. This becomes particularly critical when the nutrient composition in their environment changes. To accommodate such changes, cells have evolved specialized adaptation pathways that sense the nutrients available in their surrounding and adapt their gene expression and protein functions accordingly. It is typically assumed that these different starvation pathways have been optimized to enable growth in conditions in which nutrients are limiting. We reasoned, however, that since starvation periods are necessarily followed by nutrient retrieval, the starvation response may further function to optimize the recovery from starvation once nutrients are replenished. In the present study, we show that this is indeed the case for the well-studied phosphate starvation pathway in budding yeast. Using a combination of transcription profiling and high-resolution competition assays, we have characterized, at high temporal resolution, the growth-fitness advantage cells gain from activation of the phosphate starvation program when they enter into starvation and also when they exit starvation when nutrient is replenished. We find that the cells activate the starvation program in 2 temporal waves. The first wave is activated at intermediate depletion that is not yet limiting for growth, while the second wave is activated when phosphate becomes limiting. Of note, we provide evidence that the early response promoted recovery from starvation by increasing phosphate influx. Our results corroborate the notion that cells have evolved not only to optimize their instantaneous growth but also to prepare for future, predicted challenges.

## Introduction

Growing cells require an influx of nutrients from their environment to sustain metabolic activity. Depletion of essential nutrients arrests cell growth until the nutrients are replenished [1]. Prior to this arrest, a starvation program that regulates the expression and activity of multiple genes and proteins is activated. This starvation program increases the level of internal nutrient, e.g., by expressing high-affinity transport, mobilizing stored nutrients, or scavenging the limiting nutrient from internal or external sources [2]. This starvation program does not eliminate growth arrest but only delays its onset: a growing population will still deplete the available nutrient and arrest, but this arrest will be postponed by a limited number of generations [3,4].

Once the depleted nutrient is replenished, cells reenter the cell cycle and begin to proliferate. Return to growth is not immediate but follows a lag time during which cells adjust to the rich medium. Since growth is exponential, minimizing the lag time would provide cells with a significant competitive advantage. Indeed, subjecting cells to continuous cycles of nutrient depletion and replenishment selects for cells with accelerated recovery [5].

**Fig 1:**
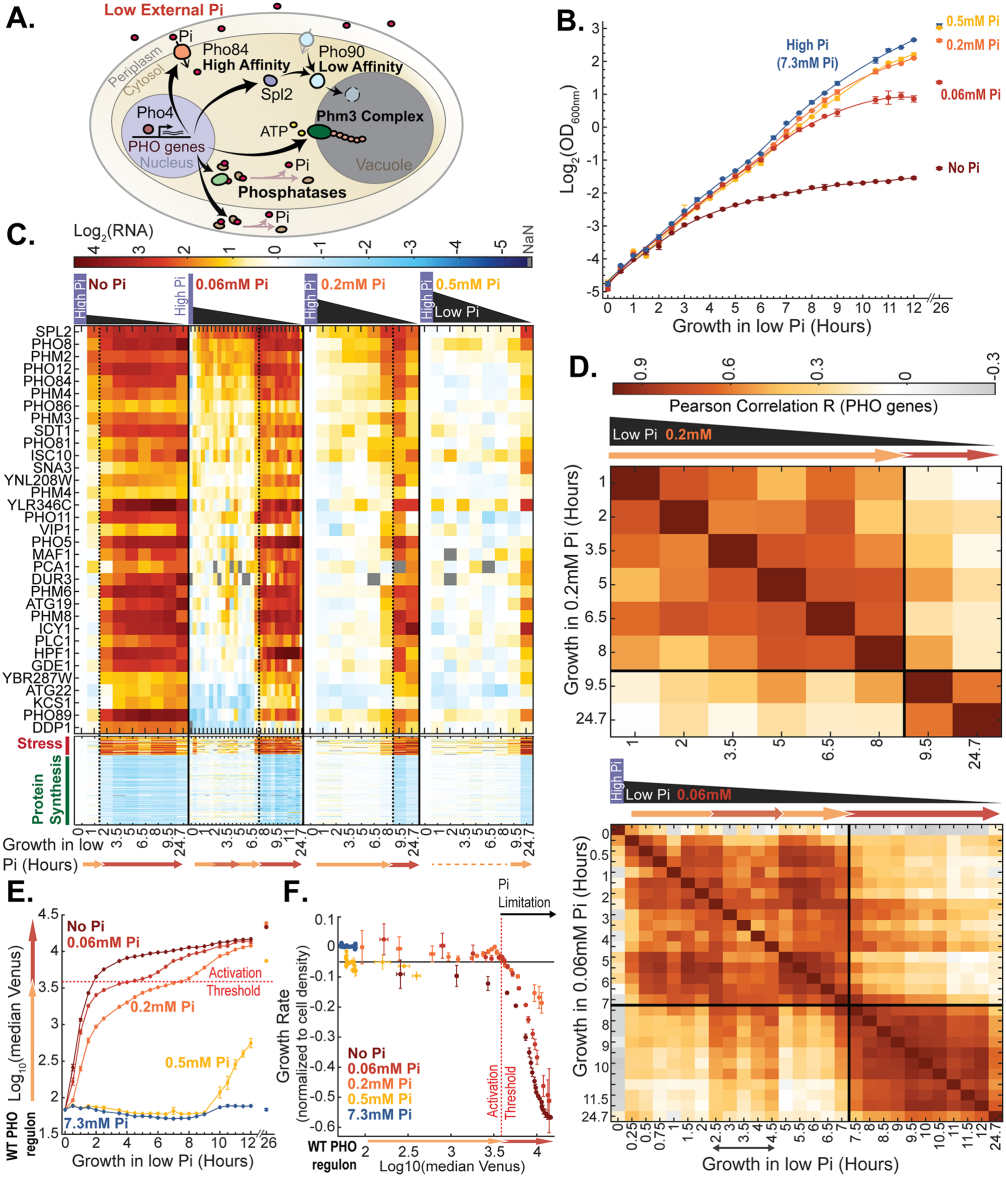
Two-wave induction of the phosphate starvation response. (A) Schematic illustration of the phosphate starvation response: Reduction in internal phosphate leads to the nuclear localization of the transcription factor Pho4 and the induction of its target genes. Genes induced by Pho4 include high-affinity transporters (e.g., *PHO84*) and an inhibitor of low-affinity transporters (*SPL2*), genes involved in phosphate mobilization in and out of vacuole storage as PolyP (e.g., *PHM3*), and phosphatases, which scavenge phosphate from molecules inside or outside the cell. (B) Cells grow for a limited number of generations following transfer to media containing different low phosphate levels: Shown is the temporal increase in cellular density (measured by optical density [OD]) following transfer to low-phosphate media, as indicated (Materials and methods). The data are the mean and standard error of 2 replicates. (C–D) Sequential induction of the phosphate transcription response: Wild-type (WT) cells were transferred from rich medium into media containing the indicated low Pi level and followed for 24.7 hours (Materials and methods). Samples for RNA sequencing were taken at the indicated time points. Shown in (C) is the log_2_(expression) change in Pho4-target genes, stress response genes (Stress), and genes coding for ribosomal proteins (Protein Synthesis), as defined in [20] (see Materials and methods and S2 Data). Data were normalized as described in the Materials and methods, and values under detectable levels (not a number [NaN]) are depicted in grey. Vertical lines indicate the second transcription wave, as defined by clustering (using k-means) of the Pearson correlation matrix, shown in (D). Note that when transferred to 0.06 mM Pi, cells transiently induced the second wave approximately 3–4.5 hours following the transfer, followed by a stable induction at approximately 7 hours after the transfer. This transient induction is likely due to the double-feedback design of the system, as we showed [21]. The reduction in growth rate (see E–F below) is observed already with first induction of the second transcription wave. The data in (C–D) are from 1 replicate. Additional biological (and experimental) replicates for growth in 0.06 mM and 0.2 mM Pi are found in Fig 3C and S1C Fig, respectively, and for 0 mM Pi, see [21]. For further supporting data for (C), see (E). See S1A and S1B Fig for definition of Pho4-target genes. (E–F) Phosphate becomes growth limiting concomitant with the induction of the second transcription wave: The PHO84p-Venus reporter is up-regulated following transfer of cells to low-phosphate media, as indicated (E). The cell growth rate was calculated by the logarithmic slope of the OD curve, shown in (B), and is plotted as a function of reporter expression (F). To control for density-dependent effects, the growth rate was normalized by that of cells transferred to rich phosphate medium (Materials and methods). The data in (E–F) are the mean and standard error of 2 replicates. Note that growth rate begins to decrease when the PHO84p-Venus reporter crosses a given threshold, independently of the incubating conditions. This crossing of the activation threshold coincides with the time at which the second transcription wave is induced, as defined by clustering (S1E Fig). See S1D Fig for the normalized growth rate as a function of time. The raw data for (B, E–F) are available in S1 Data, and those for (C–D) are in S2 Data. Pi, inorganic phosphate; WT, wild type.

We hypothesized that the starvation program activated when nutrients are depleted prepares cells not only for growth in the limiting environment but also for their subsequent recovery once nutrients are replenished. To examine this hypothesis, we focused on the phosphate starvation program in budding yeast, whose molecular basis has been characterized in detail. Cells transferred to a medium containing low phosphate induce dozens of genes. Those genes regulate intracellular phosphate levels by enabling high-affinity phosphate transport [6–8], phosphate mobilization in and out of vacuolar storage [9,10], and phosphate scavenging from different intra- and extracellular sources (Fig 1A) [11–14]. Induction of all phosphate-responsive genes depends on the transcription factor Pho4. When intracellular phosphate levels are reduced, Pho4 is dephosphorylated and enters the nucleus to activate its target genes [15–19].

We previously suggested that the phosphate starvation program promotes not only growth in phosphate-limiting conditions but also recovery from starvation, based on our analysis of cells that constitutively express *PHO84*, the high-affinity phosphate transporter [22]. When transferred to media containing low phosphate levels, the *PHO84*-constitutive cells induced the starvation program with a delay relative to wild-type cells, and when transferred back to rich media, these cells showed a longer lag time and were outcompeted by wild-type cells. Notably, the impaired recovery was rescued by partially activating Pho4 also in nonstarving cells, suggesting that optimal recovery depends on the proper, early induction of the starvation program, as seen in wild-type cells. Still, the contribution of the phosphate starvation program to cell growth has not been measured directly.

In this study, we set to quantify the contribution of the phosphate starvation program to cell growth in limited phosphate and upon recovery from starvation. We reasoned that this contribution may depend on the kinetics by which phosphate is depleted. In particular, gradual depletion of phosphate may provide cells with enough time to prepare for the upcoming limitation, activating the starvation program before arresting cell growth. We therefore considered different kinetics of phosphate depletion and followed, at high temporal resolution, the gene expression and growth rate of wild-type cells and of mutant cells that do not properly activate the starvation program. We find that cells activate the starvation program sequentially: the first activation wave is seen when phosphate is only partially depleted and before cell growth is reduced. This phase may serve as a preparation period, as deleting *PHO4* does not affect cell growth at this phase. This initial activation is followed by a second wave of expression, observed at the time when cell growth begins to slow down. At this stage, deletion of *PHO4* further reduces growth rate. Notably, we show that the early transcription wave contributes to the recovery from starvation by increasing phosphate influx once nutrients are replenished. We propose that cellular signaling is tuned not only for optimizing instantaneous cell growth but also for preparing cells for predictable future needs.

## Results

### A 2-step induction of the phosphate starvation response during growth in low-phosphate media

The phosphate starvation program in budding yeast is activated when intracellular phosphate levels are reduced. The kinetics by which the starvation program is activated therefore depends on the rate by which internal phosphate is depleted. To monitor different kinetics of phosphate depletion, we inoculated cells in media containing different low-phosphate levels, ranging from 0 mM to 0.5 mM of inorganic phosphate (Pi). Cells incubated in these media grew for a different number of generations, ranging from approximately 8 generations when incubated to 0.5 mM Pi to only approximately 3.5 generations when incubated in medium lacking phosphate (Fig 1B).

We measured the growth rate of the cells following their transfer to the low-phosphate media and profiled their transcription response at multiple time points following the transfer, as indicated (Fig 1B and 1C). To examine the starvation program, we first focused on the induction of Pho4-target genes (Fig 1C and S1A and S1B Fig). Pho4-targets were defined by comparing gene expression in cells lacking *PHO4* to that in cells that express a constitutive strong *PHO4* allele (Materials and methods) and were consistent with previous reports (S1A Fig) [9,19]. As expected, the starvation program was induced with different temporal kinetics, depending on the phosphate level in the inoculation medium. Cells transferred to a medium containing 0.5 mM Pi, for example, did not activate Pho4-target genes during the approximately 9 initial hours, showing a partial induction only at the 24.7-hour time point. By contrast, cells transferred to a medium lacking any phosphate induced Pho4-target gene expression practically immediately upon the transfer (Fig 1C).

Previous studies reported that cells transferred to medium lacking phosphate activated the starvation program in 2 sequential waves [19,21,23]. To examine whether this sequential induction is seen also in our data, we measured the similarity in the expression response at different time points following the transfer, as defined by the Pearson correlation (Fig 1D and S1C Fig). Indeed, 2 waves of transcription response were observed. The temporal delay between the 2 waves varied depending on the incubating conditions, ranging from approximately 2 hours in cells transferred to medium lacking phosphate to approximately 9.5 hours in cells transferred to medium containing 0.2 mM Pi (Fig 1C and 1D). This differential kinetics reflected the delayed induction of the second transcription wave. By contrast, the first transcription wave was induced practically immediately upon transfer to all media containing low Pi levels < 0.5 mM. The 2 waves differed primarily by the strength of gene induction. At the first wave, most (but not all) of the Pho4-target genes were moderately induced. At the second wave, the induction of these genes became stronger, and some additional Pho4-targets, not induced in the first wave, were now up-regulated (Fig 1C).

Budding yeast activate a characteristic large-scale transcription program termed the environmental stress response (ESR) when subjected to a variety of environmental stresses [24]. To examine whether this response is activated also in our data, we considered the group of stress response genes as defined in [20] (see Materials and methods). We noted that this response was not activated immediately upon transfer to low-phosphate media but was observed concomitant with the second wave of Pho4-target gene induction. Also at this time, cells down-regulated the expression of genes coding for ribosomal proteins and ribosome-associated functions (Fig 1C).

The synchronization of the stress response with the second wave of Pho4-target induction suggested to us that it is at this point that phosphate becomes growth limiting. To examine this, we monitored cell growth following the transfer. Cells maintained rapid growth rate during the initial stages of incubation, independent of the level of phosphate in the inoculation medium, but began to reduce their growth only at a later stage (Fig 1B). To comonitor growth rate and the induction of Pho4-target genes, we used cells that express a well-established reporter: *PHO84* promoter-driven Venus (PHO84p-Venus) [25]. *PHO84* is a Pho4-target gene that encodes for the high-affinity phosphate transporter. Upon transfer to low-phosphate media, *PHO84* expression is induced as part of the first transcription wave and is further up-regulated when the second transcription wave is activated (cf. Fig 1A). Induction of the PHO84p-Venus reporter was seen immediately upon transfer to low-phosphate media (<0.5 mM Pi), and its levels continued to increase in time as phosphate was further depleted, with different kinetics depending on the incubation conditions (Fig 1E). Notably, comparing cell growth rate with reporter induction, we observed that cells began to reduce their growth rate when the reporter crossed a particular activation threshold, independent of the initial low-phosphate levels into which the cells were incubated or of the time of incubation (Fig 1F). Further, this activation threshold coincided with the induction of the second wave of Pho4-target gene induction (S1E Fig). We conclude that cells induce the initial transcription wave when phosphate levels are reduced but are not yet limiting for growth. In contrast, the second transcription wave is induced when intracellular phosphate becomes growth limiting.

### Contribution of the starvation program to growth in low-phosphate media

**Fig 2:**
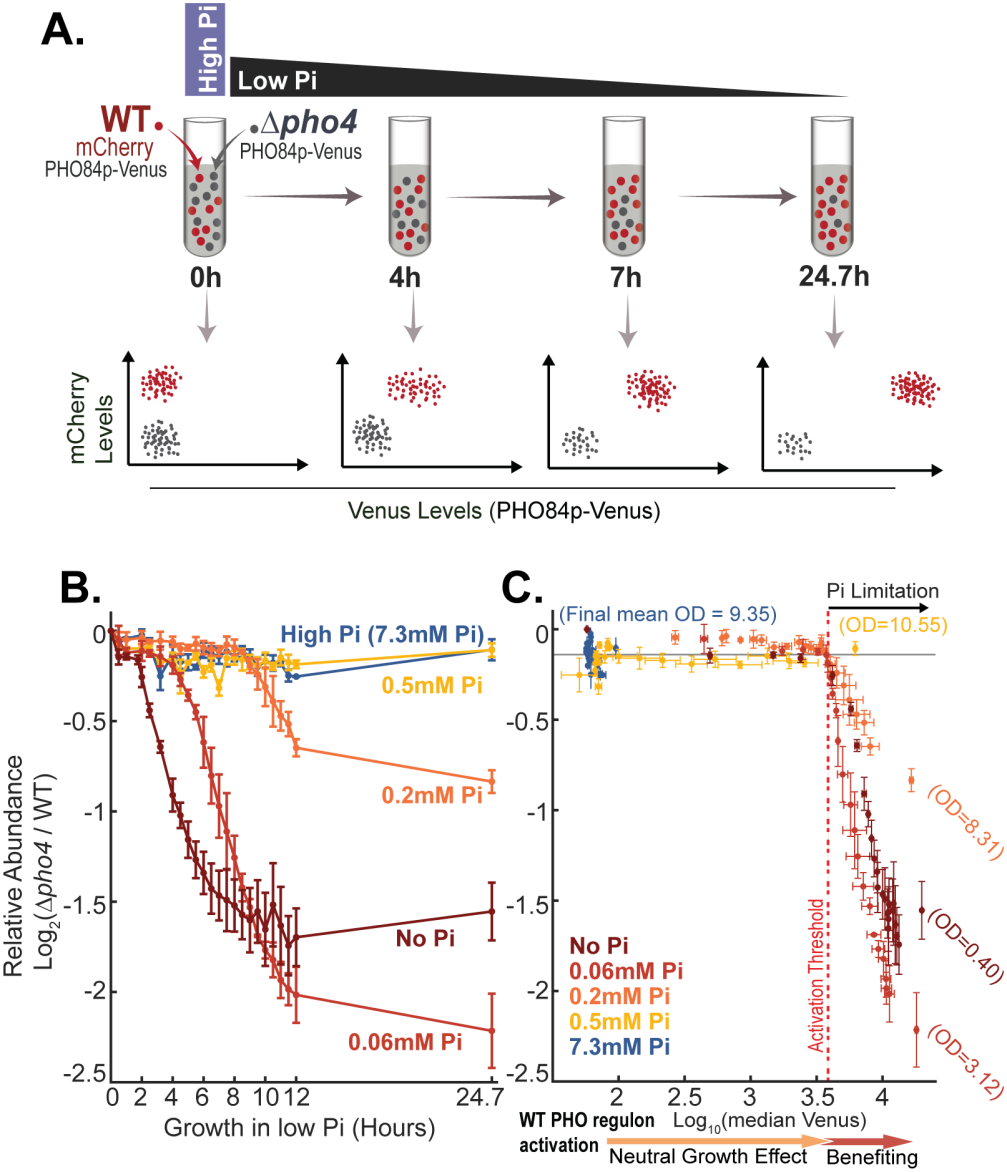
The starvation program promotes growth in low phosphate. (A) Experimental scheme: Cells deleted of *PHO4* were mixed with fluorescently labeled wild-type cells and were coincubated in different low-Pi media. Samples were taken at different times following the incubation and analyzed using flow cytometry to define the relative fraction of Δ*pho4* cells (cells not expressing mCherry) and the activation of the starvation program reporter PHO84p-Venus in individual cells. (B–C) The early phase of Pho4-target gene induction does not contribute to cell growth: The relative abundance of Δ*pho4* cells in the mixed culture is shown as a function of time (B) and as a function of the PHO84p-Venus reporter induction in wild-type cells (C). The data in (B–C) are the mean and standard error of 2 replicates. The raw data are available in S4 Data. Note that, regardless of the inoculation conditions, Δ*pho4* cells begin to reduce in frequency when the reporter level increases beyond the same activation threshold at which wild-type cells begin to reduce their growth (compare Fig 1F) and induce the second wave of Pho4-target gene expression (compare S1E Fig). See S2C–S2E Fig for the temporal induction of the reporter and a similar analysis of *Δpho81* cells. Pi, inorganic phosphate; WT, wild type.

As mentioned above, the group of Pho4-targets induced upon transfer to low-phosphate media includes multiple genes that regulate the availability of internal phosphate, including high-affinity transporters (*PHO84* and *PHO89*), genes that mobilize phosphate vacuolar storage as PolyP (PHM1-5), and phosphatases that can scavenge phosphate from other molecules inside or outside the cells (e.g., *PHO5*, *PHM8*, and *PHO11*). Induction of these genes could therefore promote growth in low-phosphate media. To quantify the contribution of this transcription program to cell growth, we considered 2 mutants that cannot activate the pathway: a strain that lacks the transcription factor Pho4 and a strain that lacks the Pho4 activating protein, Pho81. We compared the growth rate of the mutant and wild-type cells using a growth-competitive assay. For this assay, we labeled wild-type cells using a fluorescence marker mCherry, driven by the constitutively strong TEF2 promoter (Fig 2A). When mixed with unlabeled cells, the fraction of labeled (wild-type) cells in the population was measured using flow cytometry. To monitor the activation of the starvation program, we further engineered the cells to express PHO84p-Venus and monitored its induction using flow cytometry as well (Fig 2A). mCherry and/or Venus reporter expression did not detectably affect growth rate in our experimental conditions (S2A and S2B Fig).

Mutant and fluorescently labeled wild-type cells were coincubated in media containing different phosphate levels, and the fraction of wild-type cells in the population was measured periodically following this incubation, as cells grew and further depleted the phosphate (Fig 2B). Initially, the pathway-defective mutant Δ*pho4* or Δ*pho81* grew as well as wild-type cells (Fig 2B and S2D Fig). Wild-type cells began to outcompete the mutants only after a delay, which varied depending on the level of phosphate in the incubating media. For example, when incubating the cells in no-Pi or in 0.2 mM Pi, the fraction of mutant cells began to decrease only around 2 hours or around 8.5 hours after incubation, respectively.

The PHO84p-Venus reporter was induced practically immediately upon transfer of wild-type cells to low-phosphate media (<0.5 mM Pi) and continued to increase with time (S2C Fig), as described above (Fig 1E). Notably, in all conditions, the time at which the frequency of mutant cells in the mixed population began to decrease coincided with the time at which the PHO84p-Venus reporter reached a given activation threshold level, independently of the incubating conditions (Fig 2C and S2E Fig). This time coincided with the second wave of Pho4-target gene induction and the time at which wild-type cells began to reduce their growth rate (Fig 1F and S1E Fig). Therefore, induction of Pho4-target genes promotes growth in low-phosphate media, but only from the onset of the second wave of gene induction, the time at which phosphate becomes limiting for growth.

**Fig 3:**
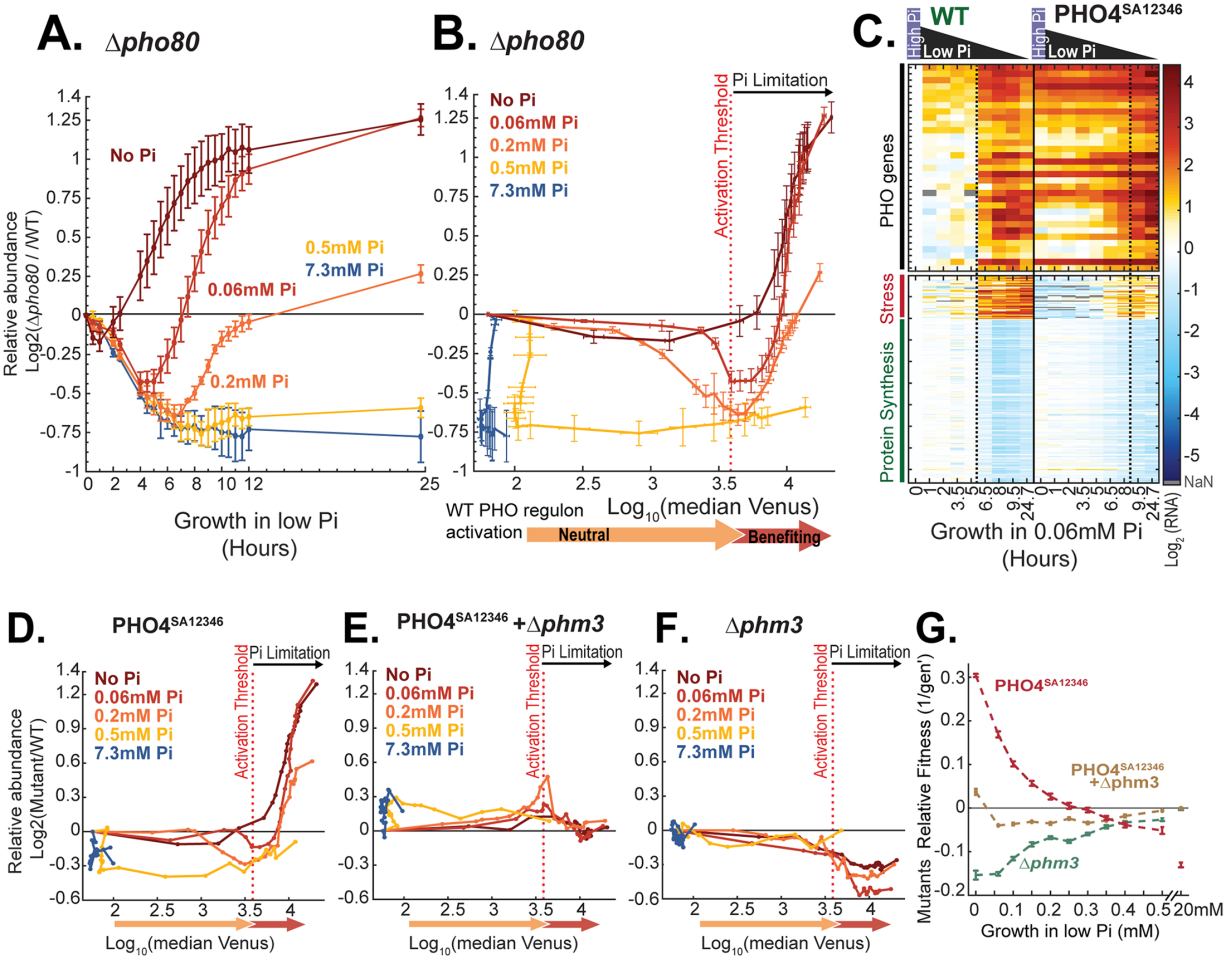
Constitutive activation of Pho4-target genes impacts cell growth. (A–D) Constitutive expression Pho4-dependent genes promote growth at low phosphate: Δ*pho80* cells and cells expressing the constitutive PHO4^SA12346^ allele were mixed with wild-type cells (either wild-type or mutant cells were mCherry labeled, respectively) and incubated in media containing different low-phosphate levels, as in Fig 2A. The (log_2_) relative abundance of Δ*pho80* cells in the mixture at different times following the incubation is shown in (A) as a function of time (see also S3D and S3E Fig for PHO4^SA12346^ and PHO4^SA1234^ cells). (B) and (D) depict the growth rate of Δ*pho80* and PHO4^SA12346^ cells as a function of reporter expression (PHO84p-Venus) in the wild-type cells, respectively. Note that, independently of the incubation conditions, the mutant cells begin to outcompete the wild-type cells when reporter activation in wild-type cells crosses the same threshold value at which wild-type cells become limiting for growth (Fig 1F), Δ*pho4* deleted cells begin to be outcompeted by wild-type cells (Fig 2C), and wild-type cells activate the second wave of Pho4-target gene expression (S1E Fig). The data in (A–B) are the mean and standard error of 3 replicates. (C) Temporal gene expression in cells expressing a constitutive Pho4 allele (PHO4^SA12346^), as in Fig 1C. Note that PHO4^SA12346^ strongly expresses the Pho4-target genes even in high Pi levels, which is consistent with previous reports [19]. The data in (C) are from 1 replicate, but see S3B and S3C Fig for related data showing equivalent behavior upon transfer into low Pi of 0.2 mM and 0.5 mM, respectively. (E–G) The growth phenotype of the Pho4-constitute strain depends on the mobilization of phosphate into storage: (E–F) same as (D), for the indicated strains. The data in (D–F) display 1 replicate; see (G) for additional supporting data. (G) The experiments in (D–F) were repeated using a variety of starvation media, and the fitness difference of mutant and wild-type cells was measured following approximately 24 hours incubation in low-Pi media. This fitness difference is shown as a function of the initial phosphate level in the starvation media. The data in (G) are the mean and standard error of 3 replicates. As described, Phm3 is a subunit of the vacuolar transporter chaperone complex, required for the storage of phosphate as polyP in the vacuoles. See S4E–S4I Fig for recovery of the above mutants from low Pi. The raw data for (A–B and D–F) are available in S4 Data. The raw data for (C) and (G) are available in S2 and S5 Data, respectively. NaN, not a number; Pi, inorganic phosphate; WT, wild type.

We next examined mutants that express the PHO regulon constitutively (expressing the PHO4^SA12346^ allele [19] or with *PHO80* deleted). Constitutive strong activation of Pho4-target genes reduced growth in rich medium and when phosphate levels were low but not yet growth limiting. By contrast, this constitutive strong activation promoted growth once phosphate became growth limiting (Fig 3A–3D and S3A–S3D Fig). Interestingly, the phenotypic effects of the PHO4^SA12346^ alleles were fully dependent on the *PHM3* gene, which mobilizes phosphate into vacuolar storage [10], suggesting that the reduced growth rates in both rich and intermediate phosphate conditions are explained by transferring too much phosphate into storage. This excessive storage promotes growth once phosphate becomes growth limiting (Fig 3D–3G).

Taken together, we find that the initial induction of Pho4-target genes occurs before phosphate becomes growth limiting and that Pho4-target induction does not contribute to cell growth during this phase. The phosphate starvation program starts contributing to growth only when phosphate becomes growth limiting, a time that is marked by the second wave of Pho4-target gene induction and up-regulation of the stress response.

### Contribution of the starvation program to recovery when phosphate is replenished

We asked whether, in addition to its role during growth in limited phosphate, the starvation program promotes recovery from starvation once phosphate is replenished. We reasoned that this contribution may depend primarily on the first wave of Pho4-target gene induction, since this wave is activated before phosphate becomes growth limiting and before it is needed for maintaining maximal growth rate. This early induction could prepare cells for upcoming starvation and further contribute to their recovery. We therefore denote the time between the induction of the first and second transcription waves as the “preparation phase.”

**Fig 4:**
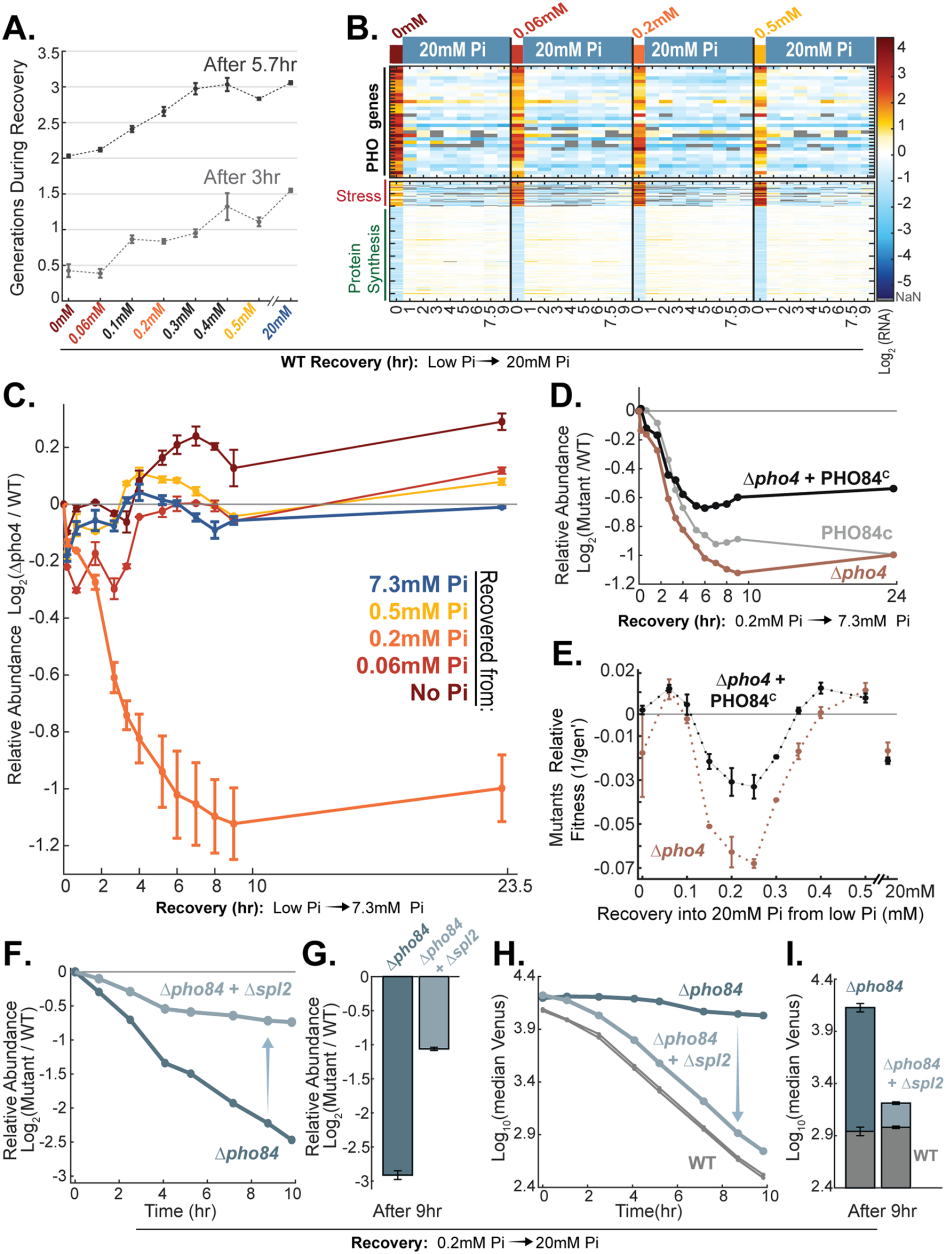
The starvation program promotes recovery once phosphate is replenished. (A) Recovery time depends on the conditions leading to starvation: Cells were incubated in media containing different low phosphate levels and maintained in the starvation conditions for 21 hours before being transferred back into a rich medium. The number of generations that cells underwent 3 or 5.7 hours after the transfer to rich medium is shown as a function of the phosphate level in the incubating media. The data in (A) are the mean and standard error of 2 replicates. (B) Pho4-target genes are rapidly down-regulated when phosphate is replenished: Recovery expression profiles of cells grown for 24.7 hours in low-Pi media (see Fig 1C) and recovered in rich medium. The data in (B) are from 1 replicate. Additional replicates for recovery from 0.2 mM and 0.06 mM Pi are found in Fig 6E and S6E Fig, respectively. See S4 Data for related experiments using a reporter gene. (C) The starvation program promotes recovery: Competition experiments, as shown in Fig 2, were performed to compare the growth of Δ*pho4* and wild-type cells following recovery in rich medium. Wild-type and Δ*pho4* cells were coincubated in media containing different levels of low phosphate, grown to saturation, and maintained in the starvation conditions for 24.7 hours before being transferred back into rich medium. The (log_2_) relative abundance of Δ*pho4* cells at different times following return to rich medium is shown. Note that Δ*pho4* cells are outcompeted by wild-type cells during recovery from starvation that was induced by incubation in media containing intermediate phosphate (e.g., 0.2 mM Pi). These conditions introduce the longest time gap between the first and second wave of Pho4-target gene induction (“preparation” time). The data in (C) are the mean and standard error of 2 replicates. See S4A Fig for similar experiments using Δ*pho81* deleted cells. (D, E) Constitutive expression of PHO84 partially rescues the recovery phenotype of Δpho4 cells: Experiments as shown in (C) were repeated for cells that constitutively express the PHO84 transporter (PHO84^C^) in a wild-type or Δ*pho4* background. The (log_2_) relative abundance of mutant cells during recovery from starvation induced by incubating in media containing 0.2 mM Pi is shown in (D). In (D), the mean of 2 replicates is shown for Δ*pho4*, and 1 replicate is shown for PHO84^C^ with and without Δ*pho4*. These data are supported by the experiments shown in (E) and Fig 5B. (E) The experiments in (D) were repeated using a variety of starvation media, and the fitness difference of mutant and wild-type cells was measured following 24.7 hours of incubation in rich medium. This fitness difference is shown as a function of the initial phosphate level in the starvation media. Note that deletion of *PHO4* impaired recovery when cells were first incubated at intermediate phosphate levels (around 0.2 mM Pi), allowing sufficient preparation time, and that constitutive expression of *PHO84* partially rescued this phenotype. The data in (E) are the mean and standard error of 3 replicates. See S4B Fig for similar experiments using Δ*pho81* cells. (F–I) Phosphate influx limits recovery from starvation: Competition experiments as in (C) were repeated for cells of the indicated genotype (F–I). Shown are the (log_2_) relative abundance of the indicated mutant strains (F–G) and the level of the PHO84p-Venus reporter in the mutants and wild-type cells (H–I) during competitive growth. In (F, H) are shown several time points within the first 10 hours of recovery (into 20 mM Pi), and those after 9 hours of recovery into 20 mM Pi are shown in (G, I). The data in (F, H) are from 1 replicate and are supported by data in (G, I), which show the mean and standard error of 2 replicates. See (S4C and S4D Fig) for recovery into a high Pi of 7.3 mM Pi. The raw data for (C, D, and F–I) are available in S4 Data. The raw data for (A, B, and E) are available in S1, S2 and S5 Data, respectively. NaN, not a number; Pi, inorganic phosphate; WT, wild type.

Cells that are incubated in media containing intermediate phosphate show a preparation phase that is prolonged relative to cells incubated in medium containing no phosphate (Fig 1C). If preparation promotes recovery, cells with prolonged preparation will recover faster than cells incubated directly in a no-phosphate medium. To examine this, we incubated cells into media containing different levels of phosphate for 21 hours, transferred them back to rich medium, and measured the lag time before growth was recovered. As predicted, the lag time decreased monotonically with increasing levels of phosphate in the incubating media (Fig 4A). In particular, cells incubated directly into medium lacking phosphate showed the longest lag time before resuming growth.

To directly examine whether the starvation program promotes recovery, we examined the Δ*pho4* and Δ*pho81* mutants that do not activate the pathway. This was done using a competition assay: fluorescently labeled wild-type cells containing the constitutive TEF2p-mCherry marker were mixed with mutant cells, incubated in low-phosphate media for 24.7 hours, and then transferred back to rich medium. The fraction of wild-type cells was measured at subsequent times following the transfer to rich medium using a flow cytometer. The gene expression profile of wild-type cells was monitored in parallel to define the kinetics by which cells down-regulate the expression of Pho4-target genes.

Replenishing phosphate rapidly repressed Pho4-target genes (Fig 4B). Still, wild-type cells resumed growth faster than pathway-deficient mutants (Fig 4C and S4A Fig). This was observed in cells that showed a prolonged preparation time (brought to starvation following incubation in media containing an intermediate level of phosphate). In fact, in cells incubated in 0.2 mM Pi, the starvation program contributed to recovery more than to growth in the low-Pi medium itself (Fig 2B versus Fig 4C and S2D Fig versus S4A Fig). By contrast, the starvation program did not detectably contribute to the recovery of cells that were incubated directly in very low or no-phosphate medium, conditions in which preparation time was limited (Figs 4C and 1C and S4A Fig). Note that when incubated in 0.5 mM Pi, the cells arrested before becoming phosphate limited (Fig 1B and S1D Fig), resulting in full dispensability of Pho4 both in the low-phosphate medium and during recovery (Figs 2B and 4C and S2D and S4A Figs). We conclude that the starvation program contributes to cell recovery from phosphate limitation, but only in conditions that introduce a significant delay between the induction of the first and second waves of Pho4-targeted genes.

### Phosphate influx is limiting during recovery in rich media

The starvation program could promote recovery from starvation by limiting phosphate toxicity. Indeed, upon recovery, cells are subject to a large, sudden influx of phosphate, which may be toxic. To examine whether phosphate toxicity limits recovery of the pathway defective Δ*pho4* or Δ*pho81* cells, we constitutively expressed the high-affinity transporter *PHO84* (PHO84^c^) in these backgrounds. We reasoned that this constitutive expression will increase phosphate uptake and toxicity, further delaying recovery from starvation. In contrast to this expectation, constitutive expression of *PHO84* did not abrogate but rather compensated for the delayed recovery of Δ*pho4* and Δ*pho81* mutants (Fig 4D and 4E and S4B Fig; see S2F and S2G Fig for growth in low Pi). This refutes the hypothesis that pathway activation is needed to prevent toxicity upon phosphate replenishment but suggests that it is required to promote phosphate influx. Indeed, to resume growth, cells must synthesize a large amount of RNA, likely requiring an increased phosphate influx.

If phosphate influx is limiting during recovery, cells without the high-affinity phosphate transporter (Δ*pho84* cells) will exit starvation with a delay. To test that, we examined the recovery time of Δ*pho84* cells (Fig 4F–4I and S4C and S4D Fig). The starvation program in Δ*pho84* cells was maintained active for at least 10 hours following phosphate replenishment, contrasting its rapid down-regulation in wild-type cells (Fig 4H–4I and S4D Fig) and confirming that the high-affinity transporter largely contributes to phosphate influx during recovery. As was expected when phosphate influx limits recovery, the growth of those cells was severely delayed (Fig 4F–4G and S4C Fig). Further, the impaired recovery of Δ*pho84* cells was rescued by deleting *SPL2* (Fig 4F–4I and S4C and S4D Fig). As one of the Pho4-target genes, *SPL2* is induced in low-phosphate media and down-regulates the activity of Pho90 (see S2H–S2J Fig for growth in low Pi). Deletion of *SPL2* therefore maintains the active status of the low-affinity transporter, leading to a higher phosphate influx, which we suggest explains the cells’ faster recovery. This recovery is still not as fast as wild-type cells, which can utilize both the low- and high-affinity transporters during recovery (Fig 4F–4I and S4C and S4D Fig).

Taken together, our results suggest that phosphate influx is limiting during the recovery of cells from phosphate starvation. Pho4-target gene induction may therefore promote recovery by facilitating the increase in internal phosphate upon recovery.

### Delayed induction of Pho4-target genes delays recovery by decreasing phosphate influx

**Fig 5:**
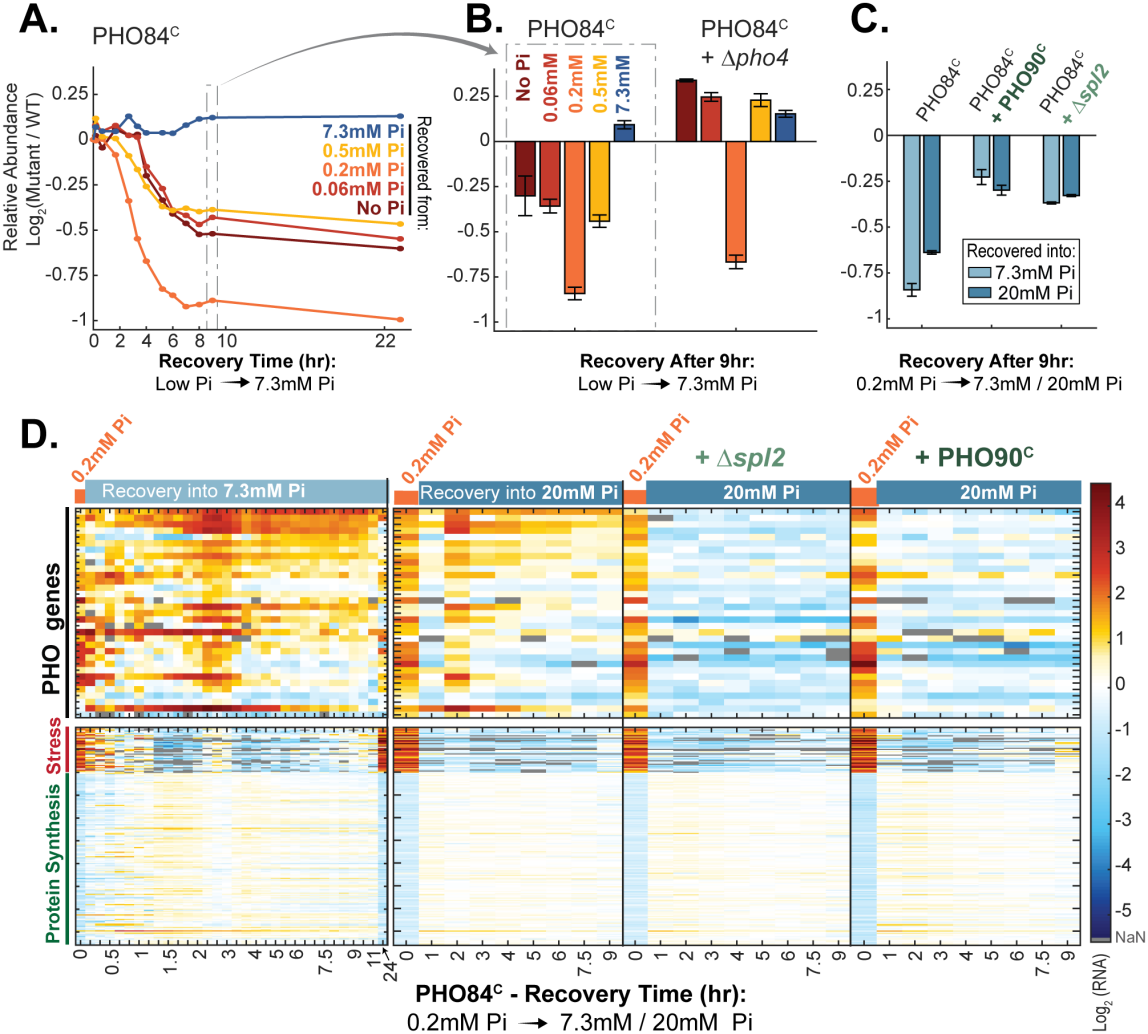
The recovery phenotype of the PHO84-constitutive strain depends on the Pho4-target gene induction. (A) Constitutive expression of the high-affinity *PHO84* transporter limits recovery from phosphate starvation: Competition experiments are the same as in Fig 4C but for cells that express *PHO84* using the constitutive *TDH3* promoter (PHO84^C^). Note that PHO84^C^ cells recover with a delay and that the delay is particularly significant when cells are recovered from a medium containing intermediate phosphate levels (0.2 mM Pi). During growth in 0.2 mM Pi, wild-type cells have a long preparation time, while PHO84^C^ triggers Pho4-target genes at a delay; see S5A Fig. The data in (A) are from 1 replicate; see (B) for additional supporting data. (B) The recovery phenotype of PHO84^C^ depends on Pho4-targeted gene expression: The experiments in (A) were repeated for PHO84^C^ and for PHO84^C^ with Δ*pho4* cells. Shown is the (log_2_) relative abundance after 9 hours of recovery into 7.3 mM Pi. The data in (B) are the mean and standard error of 3 replicates. (C–D) The recovery phenotype of PHO84^C^ is partially rescued by increasing the phosphate influx during recovery: Experiments performed as in (A) for the indicated strains and for recovery (from 0.2 mM Pi) using rich media containing a higher level of phosphate (20 mM compared to 7.3 mM Pi). PHO90^C^ denotes constitutive expression of the low-affinity transporter, while Δ*spl2* refers to the deletion of the Pho90 inhibitor *SPL2*. Both mutations were added to the PHO84^C^ strain, and the double mutants were competed against wild-type cells. The (log_2_) relative abundance after 9 hours of recovery as in (B) is shown in (C), while expression profiles of genes as in Fig 4B are shown in (D). The data in (C) are the mean and standard error of 2 or 3 replicates. The data in (D) are from 1 replicate. Additional data for recovery of PHO84^C^ strain (from 0.06 mM Pi) are found in S6E Fig. The conclusions of (D) are further supported by the Venus reporter (an independent experiment that uses an independent construct); see S4 Data. The raw data for (A–C) are available in S4 Data. The raw data for (D) are available in S2 Data. NaN, not a number; Pi, inorganic phosphate; WT, wild type.

Our analysis above mapped the contribution of the starvation program to cell recovery to the first wave of Pho4-target genes, induced before phosphate becomes limiting. Further, we showed that this contribution is pronounced only when the duration of this first wave is sufficiently long. We previously showed that constitutive activation of the high-affinity transporter *PHO84* leads to the delayed induction of the first transcription wave and shortens the delay between the 2 waves. Consistent with this first wave being important for recovery, PHO84^C^ cells recover with a delay, and this phenotype is rescued by partial activation of the starvation program using the weak PHO4^SA1234^ constitutive allele, which may mimic the first induction wave [22]. We recapitulated these results here and confirmed that delayed recovery was apparent when incubating cells in conditions that enable a long preparation phase in wild-type cells (0.2 mM Pi) but was less pronounced when preparation was short or absent (no Pi, Fig 5A and 5B and S5A–S5D Fig).

Several additional results support the hypothesis that the impaired recovery of the PHO84-constitutive cells is due to an insufficient phosphate influx upon recovery. First, the recovery phenotype was partially rescued by increased phosphate in the recovery media (20 mM compared to 7.3 mM Pi in synthetic complete (SC) medium, Fig 5C). It was also rescued by an *SPL2* deletion and by constitutive expression of the low-affinity transporter *PHO90* (Fig 5C). Further, the PHO84-constitutive cells maintained the starvation program as partially active for a considerable time following phosphate replenishment, indicating low internal phosphate. Notably, Pho4-target genes were in fact repressed rapidly upon phosphate replenishment but were also rapidly reinduced (Fig 5D). As we have shown before, this oscillatory behavior characterizes cells that are transferred from deep to intermediate internal phosphate limitation conditions [21]. This phenotype was again rescued when transferring cells into 20 mM Pi, in Δ*spl2* deleted cells and in cells that constitutively express *PHO90* (Fig 5D). We conclude that the recovery phenotype of *PHO84*-constitutive cells is due to an insufficient phosphate influx upon recovery.

### The PHO84-constitutive phenotype is alleviated by mutations that increase phosphate influx

**Fig 6:**
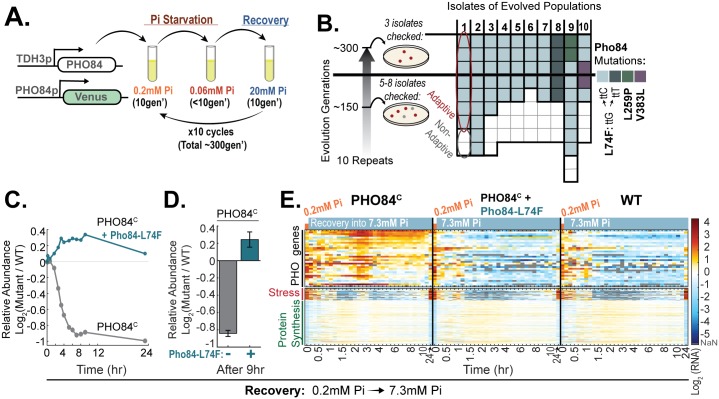
Lab evolution selects for *PHO84* mutations that rescue the recovery phenotype of PHO84^C^. (A) Experimental scheme: 16 independent lines expressing constitutively high levels of *PHO84* were subjected to 10 cycles of alternated growth in high and low phosphate for a total of 300 generations. (B) *PHO84* mutations identified in selected lines: Single-cell isolates (evolved for approximately 150 and approximately 300 generations) were selected from the 10 cultures showing improved recovery and were sent for whole-genome sequencing. All isolates showing an adaptive phenotype contained mutations in the Pho84 open reading frame (ORF), with 56 out of the 60 identified mutations mapped to the same amino-acid substitution (L74F). (C–E) Selected *PHO84* mutation rescues the recovery phenotype: The *PHO84* mutations identified in the selected stains were introduced into the wild-type allele and their effect on the growth in low Pi and on the recovery phenotype was examined when *PHO84* was expressed at wild-type levels or constitutively (both the constitutive overexpression and the evolved mutations were introduced into *PHO84* endogenous site). Shown is the recovery of the constitutive strain with and without the amino-acid substitution (Pho84-L74F(ttG/ttC)) following 24.7 hours incubation in 0.2 mM Pi (C, D) and the corresponding pho4-target gene expression during recovery (E). Note the rescue of the recovery phenotype and the immediate down-regulation of pho4-target genes. See S6B–SBJ Fig for the growth in low-phosphate media and recovery phenotypes of the selected mutations: Pho84-L74F(ttG/ttC), Pho84-L259P, and Pho84-V383L. The data in (C) are from 1 replicate; see (D) for additional supporting data. The data in (D) are the mean and standard error of 3 replicates. The data in (E) are from 1 replicate. Additional data as in (E) for recovery (from 0.06 mM Pi) are found in S6E Fig. The conclusions of (E) are further supported by experiments with the Venus reporter (see S4 Data). The raw data for (C–D) are available in S4 Data. The raw data for (E) are available in S2 Data. Gen, generations; NaN, not a number; Pi, inorganic phosphate; WT, wild type.

As a complementary approach for examining the deficiency of PHO84-constitutive cells, we subjected these cells to cycles of starvation and recovery, with the intention of selecting compensatory mutations. Sixteen independent lines were followed for approximately 300 generations, corresponding to 10 cycles of phosphate depletion and replenishment (Fig 6A). In 10 cultures, improved recovery was observed, and single-cell colonies isolated from these cultures were sent for whole-genome sequencing. Notably, all strains showing an adaptive phenotype were mutated in the *PHO84* gene. Further, 56 of the 60 individually selected cells led to the same substitution (L74F) of an amino acid located in the first transmembrane domain (Fig 6B) [6]. This mutation, as well as the 2 other amino acid substitutions identified, is found in various *PHO84* homologs (S6A Fig).

To verify the causality of the identified mutations, we introduced them into a wild-type PHO84 allele and a PHO84-constitutive allele. The impaired recovery of PHO84-constitutive cells was rescued (Fig 6C–6E and S6E–S6J Fig), and the mutation further improved the growth of both the wild-type and the constitutive strains in low-phosphate media, suggesting that it supports higher phosphate influx (S6B–S6D Fig).

## Discussion

Cells respond to the depletion of nutrients by changing the expression and activities of a large number of genes and proteins. Previous studies implicated cellular starvation programs in optimizing cell growth and survival in limiting condition [26–28]. Our results suggest that the starvation programs can also promote the recovery of cells once nutrients are replenished.

The phosphate starvation program in budding yeast was investigated extensively, revealing general principles of nutrient homeostasis [12,19,29–31]. Yet, while the molecular properties of this response are well understood, its contribution to cell growth remained relatively unexplored. Here, we closed this gap by following, at high temporal resolution, the growth of wild-type and mutant cells at different stages of phosphate depletion and recovery. We found that cells activate the starvation response before this response is required for growth. In fact, expression of Pho4-target genes is observed before cells reduce their growth rate or show any signs of stress such as expression of stress-responsive genes or down-regulating genes coding for ribosomal proteins. We propose that this initial response serves as a preparation phase, allowing cells to prepare for the upcoming limitation and for their subsequent recovery. As phosphate levels are further depleted, phosphate becomes limiting for growth, at which stage a second wave of Pho4-target genes is induced, concomitant with the induction of the general stress response. At this point, the starvation program starts contributing to cell growth. It is interesting that induction of the general stress response is delayed and becomes weaker in cells that are deleted of Pho4 and thereby do not activate the starvation program (S3A–S3C Fig), suggesting an inherent connection between this general stress response and the more specific induction of Pho4-target genes.

Most notably, we find that the phosphate starvation program also promotes recovery from starvation. As we show, recovery times might be limited by phosphate influx during the recovery, and the starvation program may promote this influx. Further, this role of the starvation program appears to depend on the early wave of Pho4-target genes, induced before phosphate becomes growth limiting.

Our results suggest that nutrient homeostasis circuits have evolved not only for promoting growth in the limiting conditions but also for preparing cells for their predictable recovery once nutrients are replenished. Recent studies suggest that this “preparation” or “anticipation” presents a general principle in microbial stress signaling. For example, the ESR is induced by moderate stresses that do not affect growth rate either in the presence or in the absence of the stress response. Thus, rather than contributing to growth in these moderate stresses, the ESR promotes survival in cases of subsequent, more severe stresses [4,24,32]. Similar anticipatory regulation was observed in other microbial systems, including examples from bacteria and yeast [33–37]. These evolved paradigms strongly suggest that throughout their natural history, yeasts encountered complex and perhaps predictable combinations of stresses so that their signaling circuits evolved to optimize not only the immediate conditions but also probably upcoming needs.

## Materials and methods

### Strains and media

*Saccharomyces cerevisiae* strains used in this study are listed in S1 Table and were created from the BY4741 background (MATa *his3Δ1 leu2Δ0 ura3Δ0 met15Δ0*) by standard genetic methods [38]. All of the relevant inserted fragments were validated by PCR followed by sequencing, except for fragments that lead to gene deletion, which were validated only by PCR. Strains fluorescently marked with constitutive cytosolic expression of mCherry were constructed from pFA6a-NAT-TEF2pr-mCherry plasmid. The amplified fragment was inserted into the HO locus by transformation. Strains fluorescently marked with constitutive cytosolic expression of GFP were constructed similarly using the pFA6aNAT-TEF2pr-GFP plasmid. Strains with the starvation response reporter PHO84p-Venus were constructed as previously described [22]. Briefly, kanMX in pBS7 (Yeast Resource Center) was replaced with the *HIS5* selection gene by BglII and EcoRI digestion from pDH5. Next, the promotor region of *PHO84* (1,000 bp upstream from the start codon) was PCR amplified from the genome with primers containing restriction sites of SalI and BamHI and inserted into pBS7 upstream of Venus. The PHO84p-Venus-HIS5 fragment was amplified using the plasmid’s canonical forward and reverse primers of pBS7 (Yeast Resource Center) with 40-bp homology upstream and downstream of HIS3 ORF for recombination into the HIS3 locus. To create a deletion of *PHO4* (nos. 5 and 7, in S1 Table), HygromycinB was amplified from the pYM-N14 plasmid [39], in which the kanMX was replaced with HygromycinB using SacI and BglII. The fragment was amplified with the following primers: AAGAGATGAGCAAAGGAGACAGAACAAGAGTAGCAGAAAGTCCGTACGCTGCAGGTCGAC and CAGTCCGATATGCCCGGAACGTGCTTCCCATTGGTGCACGGGAGCTCGTTAAAGCCTTCG. To generate the deletion of *PHO81* in the PHO84p-Venus-HIS5 strain (no. 8, in S1 Table), kanMX was amplified from pBS7 with the following primers: AATAAATGCTACATAAATGCATGCCCTTAAAACTGTATAACCATGAGATCTGTTTAGCTTGCCTCG and AATATTGATATGTAAAGTTCTGTAATATGTAGTTTTGAAATCTTAATCGATGAATTCGAGCTCG. Strains (Nos. 6, 9, and 10, in S1 Table) originated from the yeast-deletion library [40]. To create a deletion of *PHO80* in the PHO84p-Venus-HIS5 strain (no. 15, in S1 Table), the Δ*pho80*::kanMX and flanking regions were amplified from a Δ*pho80* deletion library strain [40] with the following primers: CTTCGAGAGGCAGATAACC and CCAAATGATGGCCAATTGCT. The amplified fragment was inserted instead of the *PHO80* ORF, and cells were selected on YPD +G418. Strains with partially and fully active PHO4 (PHO4^SA1234^ and PHO4^SA12346^) were constructed from previously described plasmids (EB1264 and EB1265, respectively) [19]. The amplified fragments of active PHO4, with mutations in its phosphorylation sites (serine to alanine at sites 1, 2, 3, and 4, and in PHO4^SA12346^ also proline to alanine at site 6 [19]), followed by a fused GFP and a URA3 selection, were inserted instead of the *PHO4* ORF. The addition of PHO4^SA1234^ (in strains no. 11 and no. 12, in S1 Table) was previously described [22]. To create the deletion of *PHM3* in PHO4^SA12346^ cells (no. 14, in S1 Table), *HIS5* was amplified from pDH5 with the following primers: ATTATTACTTAATTATACAGTAAAAAAAACACGCTGTGTATTCGTACGCTGCAGGTCGAC and AAATCGGCCAATAAAAGAGCATAACAAGGCAGGAACAGCTCGTTTAAACTGGATGGCGGC. To create a deletion of *PHM3* in PHO84p-Venus-HIS5 strain (no. 18, in S1 Table), CaURA3 was amplified from pAG61 with the following primers: ATTATTACTTAATTATACAGTAAAAAAAACACGCTGTGTATTGTACAGCTTGCCTCGTCC and AAATCGGCCAATAAAAGAGCATAACAAGGCAGGAACAGCTCTGATTATAATTGGCCAGTC.

Strains that constitutively express *PHO84* or *PHO90* under *TDH3* promotor (PHO84^c^ and PHO90^c^) were generated using an amplified PCR product of the *TDH3* promoter, which was inserted upstream of *PHO84* or *PHO90*. KanMX-TDH3pr was amplified from pYM-N14 [39], and a HygromycinB-TDH3pr fragment was similarly amplified from pYM-N14 in which KanMX was replaced with HygromycinB. To create a deletion of *PHO84* (no. 16, in S1 Table), kanMX was amplified from pBS7 with the following primers: ACCAGGGCACACAACAAACAAAACTCCACGAATACAATCCAAATGAGATCTGTTTAGCTTGCCTCG and TATTTGTTCTAGTTTACAAGTTTTAGTGCATCTTTGAGGCTTTTAATCGATGAATTCGAGCTCG. To create a deletion of *SPL2* (no. 20, in S1 Table), kanMX was amplified from pBS7 with the following primers: GTCACTGCAGCCACGTGCCTAGATCTATTACTATGACTTCCCATGAGATCTGTTTAGCTTGCCTCG and CAGAGGTAGAAGGTATGTATGTGTAACGATTAAGAGATGCAATCAATCGATGAATTCGAGCTCG. The double deletion of *PHO84* and *SPL2* in the PHO84p-Venus-HIS5 strain (no. 17, in S1 Table) was similarly generated using kanMX and NAT, respectively.

Phosphate-limiting media were based on SC medium, prepared from YNB without phosphate (CYN0804). Phosphate was added in the form of KH_2_PO_4_ to reach the specified levels. Fixed potassium levels were maintained by the addition of KCl (instead of KH_2_PO_4_). The media were set to a pH of 5.

### WT growth rate and reporter activation dynamics

WT cells with a starvation response reporter (Pho84promptor-Venus) and with or without mCherry were grown overnight in Erlenmeyer flasks in rich Pi medium of 7.3 mM Pi to reach logarithmic growth (OD_600_ approximately 0.4, NovaSpec Plus Visible Spectrophotometer) and were then transferred into 5 tubes. The samples were centrifuged, and all supernatant was discarded. Next, for each tube, the relevant Pi medium was added, and the samples were resuspended. The washed samples were then inoculated to the specified low- or rich-Pi medium (0 mM, 0.06 mM, 0.2 mM, 0.5 mM, and 7.3 mM Pi), and the initial OD_600_ was set to 0.05. Cells were grown in Erlenmeyer flasks at 30°C until Pi was exhausted and they reached the stationary phase (26 hours). Samples for optical density (OD_600_) and flow cytometry (to measure reporter activation levels) were taken during growth in high Pi (before wash, t = 0) and during growth in the various Pi media approximately every 30 minutes for 12 hours and at a final time point after 26 hours.

To calculate the growth rate in the different Pi media, the log_2_ (OD_600_) versus time was fitted with the MATLAB function csap (smoothing parameter of 0.4), and the values of the fitted growth curve data were determined at 1-minute intervals. The growth rate was calculated for each measured time point (between 0.5 hours and 11.5 hours) from its slope with 2 surrounding (±1 minute) fitted values. To control for density-dependent effects, the growth rate was normalized by the growth rate of cells transferred to rich phosphate medium: The growth rates in rich medium with the same optical density (OD) were subtracted from the cell’s growth rate at each time point in a given Pi medium. The growth rate used for normalization was derived from the fitted growth curve values of cells grown in rich medium (as above). The time point on the fitted growth curve with the closest OD to that of the sample was chosen for normalization. The growth rate of this time point was determined with 2 surrounding (±1 minute) values.

### Competition assays

#### Mutant relative abundance and reporter activation dynamics

All the competition experiments were performed against a WT reference strain BY4741. WT and mutant cells carried a starvation response reporter (PHO84promptor-Venus), and one of them also harbored a constitutive mCherry to distinguish between the WT and mutant cells in the flow cytometer. The PHO4^SA1234^ and PHO4^SA12346^ mutants lacked the reporter, since they also contained GFP fused to Pho4 ORF. WT and mutant cells were grown in rich Pi medium of 7.3 mM Pi to the logarithmic phase (OD_600_ approximately 0.4, NovaSpec Plus), mixed at an OD ratio of approximately 1:1, and transferred to 5 tubes. The samples were centrifuged, and all supernatant was carefully discarded. The cells were then resuspended in the relevant Pi media (0 mM, 0.06 mM, 0.2 mM, 0.5 mM, and 7.3 mM Pi) and inoculated at an OD_600_ of 0.05 in 50-ml tubes. Cells were grown at 30°C until Pi was exhausted and they reached the stationary phase (approximately 24 hours). The following day, cells were recovered in high Pi levels of 7.3 mM or 20 mM Pi as indicated, diluted to an OD_600_ of 0.05, and grown for approximately 24 hours until they reached the stationary phase. In the competition experiment of Δ*pho84* with and without Δ*spl2*, the cells were grown for 21 hours in 0.2 mM Pi (S2H–S2I Fig) and diluted to an OD_600_ of 0.01 upon recovery (Fig 4F and 4H).

The mutant/WT frequencies and the starvation response induction were established by flow cytometry. Samples for flow cytometry were taken after cells were mixed in high Pi (t = 0), at several time points during growth at low Pi levels until the stationary phase was reached (approximately 24 hours) and similarly during recovery. Approximately 50,000 events per sample were acquired by the flow cytometer. Initial events acquired by the flow cytometer were removed to reduce noise and carryover from the previous sample as follows: the acquired events were binned according to their acquisition time. A section of early acquired bins was removed only if all neighboring bins had shown a mean fluorescence deviated by more than 3 STDs from the mean. The mean was calculated from the last bins that were acquired (constituting 60% of the acquired events). The retained data constitute at least 60% of all acquired events (see S3 and S4 Data). Mutants’ (versus WT) relative abundances during growth in low Pi and during recovery were normalized to the initial time point t = 0 (prior to transfer to low Pi or prior to recovery, respectively).

#### Mutant relative fitness

The fitness was established from competition experiments preformed in 96-well plates. WT and mutant cells, marked with different fluorescent proteins, were grown overnight in 96-well flat-bottom plates in rich Pi medium of 7.3 mM Pi. The next day, the stationary cells were diluted 1/50 for 3 hours of growth in 7.3 mM Pi. WT and mutant cells were mixed at a ratio of approximately 1:1. Cells were diluted 1/10 for growth in high-Pi medium (20 mM Pi) and for flow cytometry analysis (t = 0). Prior to transfer into low-Pi media, 200 μl of mixed cells was centrifuged for 1 minute in a 96-well round-bottom plate, and 180 μl of the supernatant was discarded. Cells were resuspended with 180 μl of no-Pi medium. This wash was repeated, and the overall Pi in the medium was diluted by 1/100. Washed cells were then diluted 1/10 and grown (in 96-well flat-bottom plates) in all the relevant low-Pi media (ranging from no Pi up to 0.5 mM Pi). Therefore, cells grown in no Pi are still expected to have maximal initial Pi residues of approximately 7.3 μM Pi in their environment. After approximately 24 hours, the samples were recovered into high-Pi medium with 2 dilution steps (total of 10^−3^ dilution). The first dilution plate was read in the flow cytometer (t = approximately 24 hours in starvation, and t = 0 of recovery). After approximately 24 hours of growth in high Pi, the change in the mutant and WT ratio was measured by flow cytometry (t = approximately 24 hours of recovery). The mutants’ relative fitness was calculated for growth in Pi limitation and for the following recovery, from the slope of its relative frequencies (in log) as a function of the number of generations (based on OD measurements), divided by log(2).

#### Number of WT generations during recovery

WT cells with and without a starvation response reporter (PHO84p-Venus) were grown overnight in 50-ml tubes until reaching an OD_600_ of approximately 0.5 (NovaSpec Plus Visible Spectrophotometer). To control for the lack of fitness impact due to reporter presence, differentially labeled WT cells were mixed (WT-PHO84p-Venus was mixed with WT-mCherry, and WT-PHO84p-Venus + mCherry was mixed with WT-GFP) in a deep 96-well plate. Mixed WT cells (approximately 1:1 ratio) were transferred (240 μl) and centrifuged for 1 minute in a 96-well round-bottom plate. The supernatant was removed by flipping the plate. The cells were resuspended in the relevant low-Pi media (200 μl). This wash was repeated 2 additional times. The cells were then diluted (1/6) to an OD_600_ of approximately 0.1 (NovaSpec Plus) for growth in Pi limitations (ranging from no Pi to 0.5 mM Pi) and high-Pi medium (20 mM Pi). Cells in 96-well flat-bottom plates were grown for 21 hours in low-Pi conditions at 30°C. For recovery, cells were diluted into several plates with a high-Pi medium of 20 mM Pi (initial OD_600_ of approximately 0.1). During (and prior to) recovery, one of the diluted plates was taken both for OD measurements (OD_600nm_, Tecan Sunrise) to calculate the number of generations and for the cells’ relative growth measurements (flow cytometer) to control for a neutral fitness impact of the starvation response reporter.

### Time course experiment for RNA-seq

Cells were grown as described above for WT growth rate and reporter activation dynamics. Briefly, logarithmically growing cells (OD_600_ approximately 0.4) grown overnight in high Pi levels (7.3 mM Pi) were washed in the relevant low-Pi medium. The cells were then inoculated to a specified medium: no Pi, very low Pi (0.06 mM Pi), low Pi (0.2 mM Pi), and intermediate–low Pi (0.5 mM Pi) at an initial OD_600_ of 0.05. The cells were grown at 30°C until Pi was exhausted and they reached the stationary phase (24.7 hours). The following day, the cells were recovered into high Pi levels of 7.3 mM or 20 mM as described (diluted to an OD_600_ of 0.05) and grown for 9 hours or 24.7 hours (stationary phase). Samples for RNA sequencing were taken prior to entry into low Pi levels (t = 0) and at several time points during growth in low Pi levels until the cells reached the stationary phase at 24.7 hours; samples were taken similarly during recovery.

### RNA extraction and sequencing

The samples were centrifuged for 1 minute at 4,000 rpm, the supernatant was discarded, and the pellet was submerged immediately in liquid nitrogen and preserved in −80°C. The breaking of the cell wall was carried out in a deep 96-well plate. Cells were lysed by the addition of 450 μl of lysis buffer (1 M sorbitol (Sigma-Aldrich), 100 mM EDTA, and 0.45 μl lyticase (10 IU/μl)). The plate was incubated for 30 minutes at 30°C and then centrifuged for 10 minutes at 2,500 rpm. The supernatant was collected into a new deep 96-well plate. From this point forward, the RNA extraction was carried out using the Nucleospin 96 RNA kit, changing only β-mercaptoethanol with DTT. cDNA was prepared from the RNA extracts, barcoded, and sequenced using the Illumina HiSeq 2500 system, using Truseq SR Cluster Kit v3 -cBot-HS cluster kit and Truseq SBS Kit v3-HS run kit (50 cycles) [41].

### RNA-seq data processing and analysis

Each RNA-seq sample was mapped to the genome of *S*. *cerevisiae* (R64 in SGD) using bowtie (parameters: --best –a –m 2 –strata -5 10). Mapped reads were filtered for reads not mapped to rRNA, and the aligned filtered reads were down-sampled to 400,000 reads in order to have similar data from all samples. PCR bias was normalized for by using the unique molecular identifier (UMI), scoring each position on the genome by the unique number of UMIs it had out of the 256 possible UMIs [41]. The expression of each gene was determined by summing all of the reads aligned to the 400 base pairs upstream to its 3′ end to 200 base pairs downstream from the 3′ end. For genes with high sequence similarity in the region being summed (thus having sequences aligning as good to both genes), the reads aligned were separated to both genes according to the amount of uniquely mapped sequences. The sum of all gene expression was normalized to be 1,000,000, and genes with an expression below the threshold of 10 reads were excluded. All WT and mutant RNA data presented were normalized to logarithmic growing WT cells in 7.3 mM Pi (t = 0 of experiment 1; see S2 Data) before the cells were distributed between no Pi, 0.06 mM, 0.2 mM, and 0.5 mM Pi and grown for 24.7 hours and recovered into 20 mM Pi. Stress and protein synthesis genes were not presented in the figures if (1) during growth in low Pi, they were below detection levels (not a number [NaN]) in the (normalized) start time point (t = 0, 7.3 mM Pi) or (2) during recovery from low Pi, they were below detection levels (NaN) in the (normalized) t = 0 time point of recovery (the last time point of growth in low Pi prior to recovery).

### Classification of PHO-regulated genes, stress genes, and protein synthesis genes

Genes were classified as PHO-regulated genes if they met 2 RNA expression criteria. The first required up-regulation in ON cells (PHO4^SA12346^) versus OFF cells (Δ*pho4*) in at least 15 out of 25 samples. The samples (3*8) were taken during growth in low Pi of 0.06 mM, 0.2 mM, and 0.5 mM Pi (time points: 1, 2, 3.5, 5, 6.5, 8, 9.5, 24.7 hours), and 1 sample was taken from logarithmically growing cells in high-Pi medium (prior to the transfer to the different low-Pi media). Genes were considered up-regulated if the expression ratio between ON and OFF cells was larger than or equal to 2 or if the OFF cells were below detection levels (NaN) and the ON cells were 10 times higher than the defined RNA detection level (10 reads). The second criterion required up-regulation in WT cells during growth in no Pi medium in at least 6 out of 8 time points (1, 2, 3.5, 5, 6.5, 8, 9.5, and 24.7 hours). Genes were considered up-regulated if the expression ratio between no Pi and high Pi levels was larger than or equal to 2. The sample grown in high-Pi medium was taken during logarithmic growth in 7.3 mM Pi, prior to transfer into no Pi. Genes that were below detection levels in high Pi were removed from the analysis. Stress response genes (Stress) and genes coding for ribosomal proteins (Protein Synthesis) were defined in [20] (see S2 Data for gene names). Those transcriptional modules were characterized based on coexpression in over 1,000 expression profiles of *S*. *cerevisiae* during growth in diverse environmental conditions. The stress module we have used (46 genes) is a subgroup of the ESR [24].

### Flow cytometry

Flow cytometry was done with a BD LSRII system (BD Biosciences). GFP and Venus samples were analyzed with excitation at 488 nm and emission at 525 ± 25 nm. Samples with mCherry were analyzed with excitation at 594 nm and emission at 610 ± 10 nm.

### Experimental evolution

A PHO84^C^ strain marked with mCherry and with a reporter of the starvation response (PHO84p-Venus) was evolved in the lab to create 16 independent populations. The experimental evolution was performed in 50-ml tubes, with a volume of 10 ml. Stationary cells grown in high-Pi (20 mM Pi) medium were transferred (10 μl) into 16 tubes with low Pi (0.2 mM Pi) and grown until they reached the stationary phase (an OD_600_ of approximately 8.5; cells underwent approximately 10 generations). Next, cells were transferred (10 μl) in parallel from each tube to very low-Pi (0.06 mM Pi) medium. In order to reach prolonged Pi starvation, cells were grown for at least 2 days, reaching a final OD_600_ of approximately 4 and undergoing approximately 9 generations. Then, 25 μl of stationary cells from each tube was recovered into a 20 mM Pi medium and grown until stationary phase (an OD_600_ of approximately 9.5, cells underwent approximately 10 generations). This cycle of fluctuating Pi levels was repeated 10 additional times, for a total of approximately 300 generations of evolution. Before each transfer, OD_600_ was measured and PHO84p-Venus levels were verified by flow cytometry. Samples were frozen for further analysis throughout the evolution.

### Whole genome sequencing of evolved isolates

Whole genome sequencing was performed to identify the adaptive mutations in evolved isolates. To this end, the ancestor and 60 isolates from 10 evolved populations were sequenced. The isolates were taken from the middle (after approximately 150 generations, 30 isolates) and the end of a lab-evolution experiment (after approximately 300 generations, 30 isolates). The fitness of the isolates was verified by a competition assay. Only isolates in which the limited recovery of the ancestor (PHO84^C^) cells was rescued were sequenced. All of the isolates after approximately 300 generations were adapted, while after approximately 150 generations, only 30 out of the 56 isolates were adapted. DNA was extracted from 2 ml of saturated overnight culture grown in YPD. The cells were pelleted, resuspended in 10 ml sorbitol 1M at 4°C, and centrifuged for 2 minutes at 4°C at 4,000 rpm, and the supernatant was discarded. The cells were then resuspended in 300 μl of lysis buffer (50 mM HEPEs pH 7.5, 140 mM NaCl, 1 mM EDTA, 1% TritonX-100, and 0.1% sodium deoxycholate) and transferred to 2-ml safe-lock Eppendorf tubes with 0.5-mm Zirconium Oxide beads. They were then blended in a Bullet Blender 24 (Next Advance) for 1 minute at level 8. The supernatant was transferred to new 1.5-ml tubes together with an additional 200 μl of lysis buffer used to wash the beads. Lysates were treated for 60 minutes with RNAse at 37°C and then treated for an additional 2 hours with Proteinase K (20 mg/ml) at 37°C. Cleared lysates were sonicated for 30 minutes (30 seconds on, 30 seconds off) in a Bioruptor plus (Diagenode) cooled water bath sonicator, resulting in an average DNA fragment size of approximately 300 bp. Ten ul of the lysate was taken from each sample, and a multiplexed library was prepared for sequencing [42]. The library was sequenced in an Illumina HiSeq 2500 with 100-bp paired-end sequencing.

### Genotyping

DNA reads from the different isolates were aligned to the yeast genome against the S288C_reference_sequence_R64-1-1_20110203_edited.fa S288C reference genome using bwa (ver. 0.6.2) [43]. Duplicated reads were removed with picard-tools (ver. 1.73), and the bam files of the samples were merged. Determination of SNP positions was performed using the genome analysis toolkit (GATK) Unified Genotyper [44]. Since there was no SNP database available, a database was created from our data. Towards this task, the following GATK (ver. 2.2–16) tools were used on the merged bam file: RealignerTargetCreator, IndelRealigner, and UnifiedGenotyper (-stand_call_conf 50.0 -stand_emit_conf 20.0 --sample_ploidy 1 --genotype_likelihoods_model BOTH --max_alternate_alleles 1 -dcov 100); from this first round of variant calling, the variants were filtered using SelectVariants (-select "QD > 10.0 && QUAL>50 && DP>10"). The vcf result file was used for the second round of variant identification. In this round, the following GATK tools were used: RealignerTargetCreator, IndelRealigner, BaseRecalibrator, and UnifiedGenotyper (with the above parameters except for --max_alternate_alleles 4 and –dbsnp). SNP annotation was added with the tool snpEff [45].

### The natural amino-acid variety in *PHO84* homologs

The amino-acid variety in *PHO84* homologs was established with ConSurf [46]. Starting from the *PHO84* protein sequence (SGD) as input, multiple sequence alignment was built using MAFFT. The homologs were collected from UNIREF90. CS-BLAST was used for the homolog search algorithm, with search parameters of CSI-BLAST E-value of 0.0001, a maximal ID of 95%, and a minimal ID of 35%, and 3 iterations of CSI-BLAST. CSI-BLAST resulted in 501 sequences, which were used to calculate the residue variety (in percentage) for each position in the query sequence (Pho84 ORF).

### Allele swapping

To create strains with the evolved amino-acid substitutions (nos. 22–25, in S1 Table), each of the 4 base substitutions evolved in the *PHO84* ORF during experimental evolution was inserted into the ancestor strain and the WT (with PHO84p-Venus reporter) in 2 transformation steps. First, *PHO84* ORF was disrupted by CaURA3 insertion, which was lifted from pAG60 [47] with the primers ATGACGAAGGTTTCGGTTGGCAACAAGTTAAGACCATCTCCTGTTTAGCTTGCCTTGTCC and CCAGTAACCAGGTAATGAACCAGCACAAATCAAAATCAGATTCGACACTGGATGGCGGCG. Next, we lifted each of the 4 evolved alleles by PCR with the primers AACATGGCCTTCCACAACC and GTTTATGGTATGCGAAACCG from representative evolved isolates. Each of the 4 amplified fragments was inserted in place of the *PHO84* deletion created above. All insertions were verified by PCR and sequencing.

## Supporting information

S1 Fig Sequential induction of Pho4-target genes. (A) Pho4-targets defined in our data: Pho4-target genes were defined as genes which are (1) induced over two-folds in a no-phosphate medium, and (2) induced over two-folds in cells expressing a constitutive PHO4^SA12346^ allele, compared to Δ*pho4* cells (Materials and methods). 33 genes fulfilled both criteria, as is shown in the Venn diagram. This set largely overlapped the PHO regulon defined previously [9,19], as shown. (B-C) Temporal induction of pho4-dependent genes: (B) Shown is the gene expression data used to generate the correlation matrix in Fig 1D. Plots with a high-temporal resolution (same data as in Fig 1C), while highlighting the ordering of genes based on their mean induction in the two hours following transfer (indicated on the left) into 0.06 mM Pi. The same order is kept throughout the manuscript. The data in (B) are from 1 replicate, for an additional biological (and experimental) replicate see Fig 3C. (C) Same as Fig 1C for additional gene expression data of wild-type cells during growth in 0.2 mM Pi. Shown is the Pearson correlation matrix for PHO genes (as in Fig 1D), and for all expressed genes. The data in (C) are from 1 replicate, for an additional biological (and experimental) replicate see Fig 1C. (D-E) Phosphate becomes growth limiting concomitant with the induction of the second transcription wave: Growth rate data shown in Fig 1F is shown in (D) as a function of time. The data in (D) are from 2 replicates. In (E), the average expression of genes induced at the second transcription wave (lowest-scoring eight genes in (B)) is shown as a function of the PHO84p-Venus reporter expression (data from the experiment in Fig 1F). Shown are time points: 1, 2, 3.5, 5, 6.5, 8, and 9.5 hours during growth in low Pi media. Note that the induction of the second wave of Pho4-target genes coincides with the crossing of the reporter activation threshold, as defined in Fig 1F. Per each of the 4 low Pi media 1 replicate is shown in (E). The raw data for (E) are available in S1 and S2 Data. The raw data for (A-C) are available in S2 Data. The raw data for (D) are available in S1 Data.
(TIF)

S2 Fig Phosphate becomes growth limiting concomitant with the induction of the second transcription wave. (A-B) Fluorescence labeling using TEF2-mCherry, or PHO84p-Venus reporter expression, does not affect growth fitness: Shown are the results of competition between wild-type strains with or without the indicated reporters. In (A), wild-type strains of the indicated backgrounds were co-incubated in media containing different levels of phosphate, as indicated, and their relative fitness was measured following approximately 24 hours of incubation (Materials and methods). Per each of the 10 low and high Pi media, 1 replicate is shown. (B) The same as Fig 2B but using a wild-type rather than Δ*pho4* cells. Per each of the 5 low and high Pi media, 1 replicate is shown. (C) Temporal induction of the starvation program: Data from the experiments in Fig 2B and 2C. The cells expressed the *PHO84p*-Venus reporter whose temporal expression, as defined by a flow-cytometer, is shown. The data in (C) are the mean and the standard error of 2 replicates. For *Δpho4* gene expression profiles in low Pi levels see (S3A–S3C Fig). (D-E) The early phase of Pho4-target gene induction does not contribute to cell growth: same as Fig 2B and 2C for *Δpho81* cells. Per each of the 5 low and high Pi media, 1 replicate is shown. See Fig 2B and 2C for similar PHO regulon deficient mutant (*Δpho4*) cells. (F-J) Limited phosphate transport is a main cause for the reduced growth of *Δpho4* cells when entering a limited phosphate regime: Similarly as Fig 2C, for the indicated strains and the indicated conditions (F-I). PHO84^c^ denotes a strain that expresses the *PHO84* high affinity transporter constitutively using the strong *TDH3* promoter; *Δpho84* is a strain deleted of *PHO84*, while *Δpho84+Δspl2* indicates a strain deleted also of *SPL2*, the inhibitor of the low affinity transporter Pho90 that, similarly to *PHO84*, is also induced by Pho4. In (J) shown (log_2_) relative abundance of cells grown in low Pi medium till deep stationary phase. Samples were taken after 21–24.7 hours in low Pi (0.2 mM Pi). Note that increased activity of the low affinity transporter compensates for the lack of *PHO84*, and that the double deleted strain (*Δpho84+Δspl2*) phenotype resembles that of the *Δpho4* strain. The data in (F-G) are the mean and standard error of 2 to 3 replicates (Δ*pho4* and Δ*pho4*+PHO84^C^, respectively). The data in (H-I) are the mean and standard error of 2 replicates (Δ*pho4*) and 1 replicate (*Δpho84* with and without *Δspl2*). This data is supported by the experiments shown in (J). The data in (J) are the mean and standard error of 2 replicates (Δ*pho4*) and 3 replicates (*Δpho84* with and without *Δspl2*). The raw data for (B) are available in S3 Data. The raw data for (C-J) are available in S4 Data. The raw data for (A) are available in S5 Data.
(TIF)

S3 Fig Constitutive activation of Pho4-target genes affects cell growth. (A-C) Gene expression profiles of cells expressing the constitutive PHO4 allele: Same as Fig 1C for the indicated strains. PHO4^SA12346^ is a strong activating allele, while PHO4^SA1234^ is a weak activating allele [19]. *Δpho4* is a PHO regulon deficient mutant. The data acquired during growth in 0.06 mM Pi (A), 0.2 mM Pi (B), and 0.5 mM Pi (C) are from 1 replicate. See (Fig 3C) for PHO4^SA12346^ growth in 0.06 mM Pi. Notice, constitutive PHO4 allele strains ability to strongly (PHO4^SA12346^) or partially (PHO4^SA1234^) express the Pho4-target genes even in high Pi levels (prior to transfer into low Pi, t = 0), as shown previously [19]. This activation is maintained also during the initial hours of growth in low Pi. The absence of the regulon induction seen in *Δpho4* (A-C) was also verified with a Venus reporter (an independent experiment that uses an independent construct) (see S2C Fig). (D-E) Constitutive expression of Pho4-depdent genes promotes growth in low phosphate: Same as Fig 3A for the indicated strains. Per each of the five low and high Pi media in (D-E) 1 replicate is shown. The data in (D) are supported by Fig 3G. The raw data for (D-E) are available in S4. The raw data for (A-C) are available in S2 Data.
(TIF)

S4 Fig The starvation program promotes recovery once phosphate is replenished. (A) The starvation program promotes recovery: Same as Fig 4C for *Δpho81* cells. The data in (A) displays 1 replicate (per each of the 5 low and high Pi media) and is supported by the experiments shown in (B). (B) Constitutive expression of PHO84 partially compensates for the recovery phenotype of *Δpho81* cells: same as Fig 4E for the indicated strains. The data are the mean and standard error of 3 replicates. (C-D) Both the low and high-affinity transporters are required for recovery from starvation: Same as Fig 4G and 4I for recovery into high Pi (7.3 mM Pi). The data in (C-D) are the mean and standard error of 2 replicates. (E-I) Strong constitutive activation of Pho4-target genes impedes recovery: (E-G) same as Fig 4C for the indicated strains. PHO4^SA1234^ and PHO4^SA12346^ are the weak and strong activating PHO4 alleles, respectively [19]. The data in (E-G) displays 1 replicate (per each of the five low and high Pi media). Data in (F) is supported by the experiments shown in (I). (H) Expression profiles same as Fig 4B for the indicated strains during recovery. The data are from 1 replicate. (I) *Δphm3* partially compensates for the recovery phenotype of constitutive activation of the PHO4^SA12346^ allele: same as Fig 4E for the indicated strains. The data in (I) are the mean and standard error of 3 replicates. The raw data are available in S5 Data. The raw data for (A, C-G) are available in S4 Data. For (H) the raw data are available in S2 Data.
(TIF)

S5 Fig Recovery phenotype of PHO84-constitutive depends of Pho4-target gene induction. (A) The induction of Pho4-target genes reporter is delayed in PHO84^C^ cells: wild-type and PHO84^C^ cells expressing PHO84p-Venus reporter were mixed and transferred to media containing different levels of phosphate, same as S2C Fig. Cells were followed for twelve hours. Shown is the reporter expression in the 2 strains as a function of incubation time. The data in (A) are the mean and standard error of 3 replicates. (B) Induction of early wave of Pho4-target genes is delayed in PHO84^C^ cells: Same as Fig 1C for the indicated strains. The data in (B) are from 1 replicate and is supported by Venus reporter experiments shown in (A). (C-D) Weak constitutive activation of Pho4-target genes rescue recovery of PHO84^C^ cells: same as Fig 4C for the indicated strains. PHO4^SA1234^ is the weak *PHO4* allele. Cells were recovered into high Pi of 20 mM Pi after 23 hours in the indicated low Pi levels. The data in (C-D) displays 1 replicate (per each of the five low and high Pi media), and supports previous report [22]. The raw data for (A, C-D) are available in S4 Data. The raw data for (B) are available in S2 Data.
(TIF)

S6 Fig Lab evolution selects for PHO84 mutations that rescue the recovery phenotype of PHO84^C^. (A) Lab evolution selected for mutations that appear in PHO84 homologs: shown are the 3 amino-acid substitutions (Pho84-L74F, Pho84-V383L, and Pho84-L259P) identified in 60 evolved isolates, as described in Fig 6B and their presence in PHO84 homologs from different species (Materials and methods).The natural polymorphism of leucine to proline (L259P), is highly conserved and was previously suggested to be adaptive during phosphate starvation [48,49] (B-C) The selected PHO84 mutation promotes growth in low Pi: (B) Same as Fig 2B following transfer to a medium containing 0.06 mM Pi for the indicated strains. The *PHO84* mutations (L74F (ttG/ttC), V383L, and L259P) identified in the selected stains (see Fig 6B) were introduced into wild-type and PHO84^C^ cells instead of the wild-type allele (in PHO84^C^ cells both the constitutive promoter and the evolved mutations were introduced into *PHO84* endogenous site). The data in (B) are from 1 replicate and is supported by the experiments shown in (C). In (C) shown is the (log_2_) relative abundance after 24.7 hours of growth in the indicated low and high Pi levels. The data in (C) are the mean and standard error of 3, 2, or 1 replicates (see bar edge color: black, grey, or none, respectively). (D) The Pho84-L74F alters the temporal induction of Pho4-target genes: transcription profiles, as in Fig 1C, following transfer to a medium containing 0.06 mM Pi for the indicated strains. The data in (D) are from 1 replicate and is supported by the experiments with a Venus reporter (see S5A Fig and D4 Data). (E-J) Selected *PHO84* mutation rescues the recovery phenotype: Same as Fig 6C–6E for the indicated mutations and starvation conditions. The data in (E) are from 1 replicate and is supported by the experiments with a Venus reporter, see D4 Data. The data in (F-I) are from 1 replicate and is supported by the experiments shown in (J). the data in (C) are the mean and standard error of 3, 2, or 1 replicates (see bar edge color: black, grey, or none; respectively). The raw data for (B, C, F-J) are available in S4 Data. The raw data for (D, E) are available in S2 Data. The raw data for (A) are available in S6 and S7 Data.
(TIF)

S1 Table List of strains used in this study. (XLSX)

S1 Data WT growth rate in low Pi, Venus reporter activation dynamics in low Pi, and number of generations during recovery. (XLSX)

S2 Data RNAseq data. (XLSX)

S3 Data Competition assay: Relative abundance of WT control. (XLSX)

S4 Data Competition assays: Mutant relative abundance and Venus reporter activation dynamics. (XLSX)

S5 Data Competition assays: Mutant relative fitness. (XLSX)

S6 Data FASTA protein sequences used to establish the natural amino-acids variety in PHO84 homologs. (TXT)

S7 Data The amino-acids variety in PHO84 homologs. (XLSX)

## Abbreviations

ESR: environmental stress response
NaN: not a number
OD: optical density
Pi: inorganic phosphate
SC: synthetic complete
