# Supplementary figures and images for: Dual role of starvation signaling in promoting growth and recovery
Source: PLoS Biol. 2017 Dec 13;15(12):e2002039. doi: 10.1371/journal.pbio.2002039 (PMC5728490; doi:10.1371/journal.pbio.2002039)

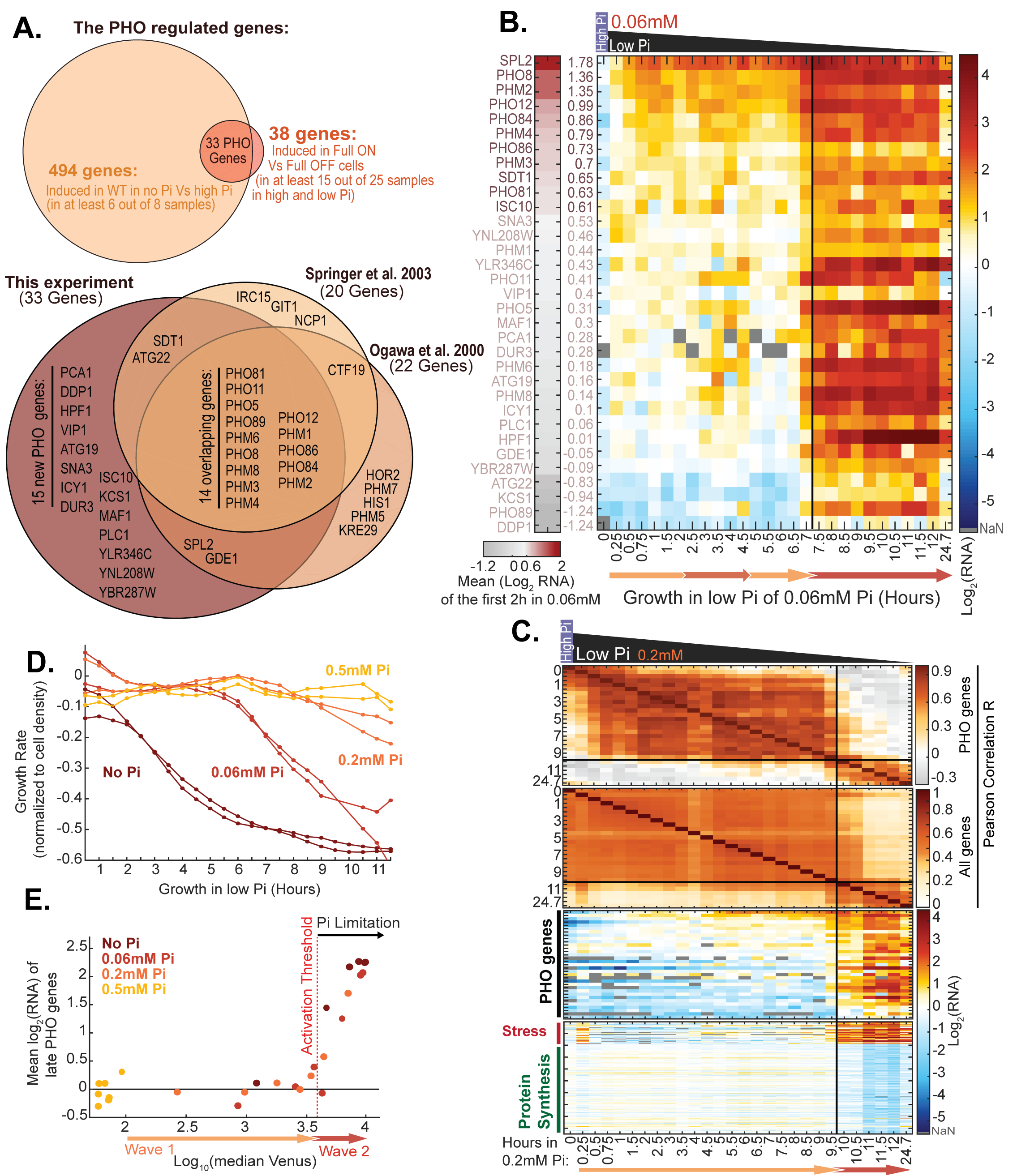

Supplement: S1 Fig — (A) Pho4-targets defined in our data: Pho4-target genes were defined as genes which are (1) induced over two-folds in a no-phosphate medium, and (2) induced over two-folds in cells expressing a constitutive PHO4SA12346 allele, compared to Δpho4 cells (Materials and methods). 33 genes fulfilled both criteria, as is shown in the Venn diagram. This set largely overlapped the PHO regulon defined previously [9,19], as shown. (B-C) Temporal induction of pho4-dependent genes: (B) Shown is the gene expression data used to generate the correlation matrix in Fig 1D. Plots with a high-temporal resolution (same data as in Fig 1C), while highlighting the ordering of genes based on their mean induction in the two hours following transfer (indicated on the left) into 0.06 mM Pi. The same order is kept throughout the manuscript. The data in (B) are from 1 replicate, for an additional biological (and experimental) replicate see Fig 3C. (C) Same as Fig 1C for additional gene expression data of wild-type cells during growth in 0.2 mM Pi. Shown is the Pearson correlation matrix for PHO genes (as in Fig 1D), and for all expressed genes. The data in (C) are from 1 replicate, for an additional biological (and experimental) replicate see Fig 1C. (D-E) Phosphate becomes growth limiting concomitant with the induction of the second transcription wave: Growth rate data shown in Fig 1F is shown in (D) as a function of time. The data in (D) are from 2 replicates. In (E), the average expression of genes induced at the second transcription wave (lowest-scoring eight genes in (B)) is shown as a function of the PHO84p-Venus reporter expression (data from the experiment in Fig 1F). Shown are time points: 1, 2, 3.5, 5, 6.5, 8, and 9.5 hours during growth in low Pi media. Note that the induction of the second wave of Pho4-target genes coincides with the crossing of the reporter activation threshold, as defined in Fig 1F. Per each of the 4 low Pi media 1 replicate is shown in (E). The raw data for (E) a [file pbio.2002039.s001.tif]

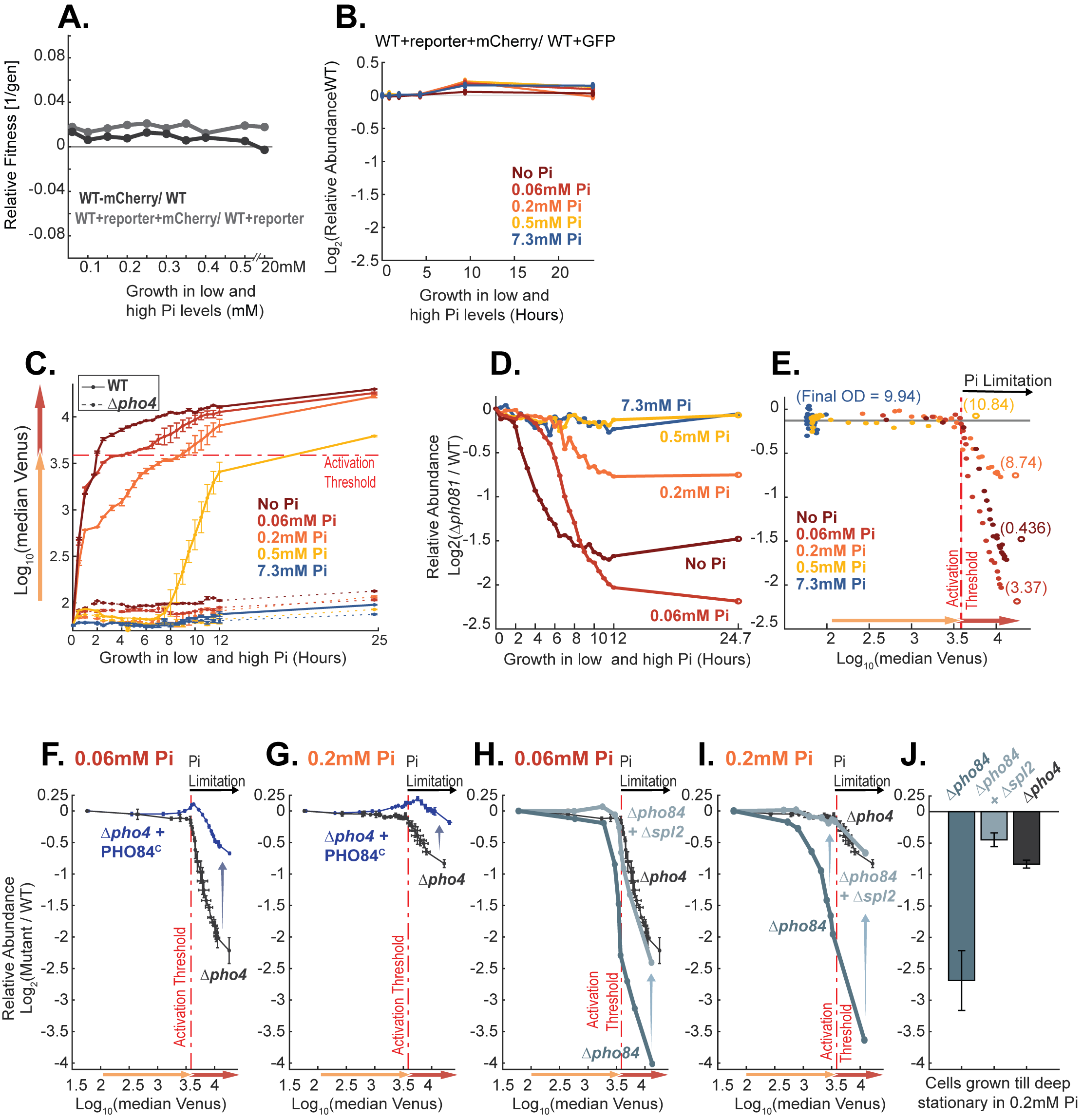

Supplement: S2 Fig — (A-B) Fluorescence labeling using TEF2-mCherry, or PHO84p-Venus reporter expression, does not affect growth fitness: Shown are the results of competition between wild-type strains with or without the indicated reporters. In (A), wild-type strains of the indicated backgrounds were co-incubated in media containing different levels of phosphate, as indicated, and their relative fitness was measured following approximately 24 hours of incubation (Materials and methods). Per each of the 10 low and high Pi media, 1 replicate is shown. (B) The same as Fig 2B but using a wild-type rather than Δpho4 cells. Per each of the 5 low and high Pi media, 1 replicate is shown. (C) Temporal induction of the starvation program: Data from the experiments in Fig 2B and 2C. The cells expressed the PHO84p-Venus reporter whose temporal expression, as defined by a flow-cytometer, is shown. The data in (C) are the mean and the standard error of 2 replicates. For Δpho4 gene expression profiles in low Pi levels see (S3A–S3C Fig). (D-E) The early phase of Pho4-target gene induction does not contribute to cell growth: same as Fig 2B and 2C for Δpho81 cells. Per each of the 5 low and high Pi media, 1 replicate is shown. See Fig 2B and 2C for similar PHO regulon deficient mutant (Δpho4) cells. (F-J) Limited phosphate transport is a main cause for the reduced growth of Δpho4 cells when entering a limited phosphate regime: Similarly as Fig 2C, for the indicated strains and the indicated conditions (F-I). PHO84c denotes a strain that expresses the PHO84 high affinity transporter constitutively using the strong TDH3 promoter; Δpho84 is a strain deleted of PHO84, while Δpho84+Δspl2 indicates a strain deleted also of SPL2, the inhibitor of the low affinity transporter Pho90 that, similarly to PHO84, is also induced by Pho4. In (J) shown (log2) relative abundance of cells grown in low Pi medium till deep stationary phase. Samples were taken after 21–24.7 hours in low Pi (0.2 mM Pi). Note that increased ac [file pbio.2002039.s002.tif]

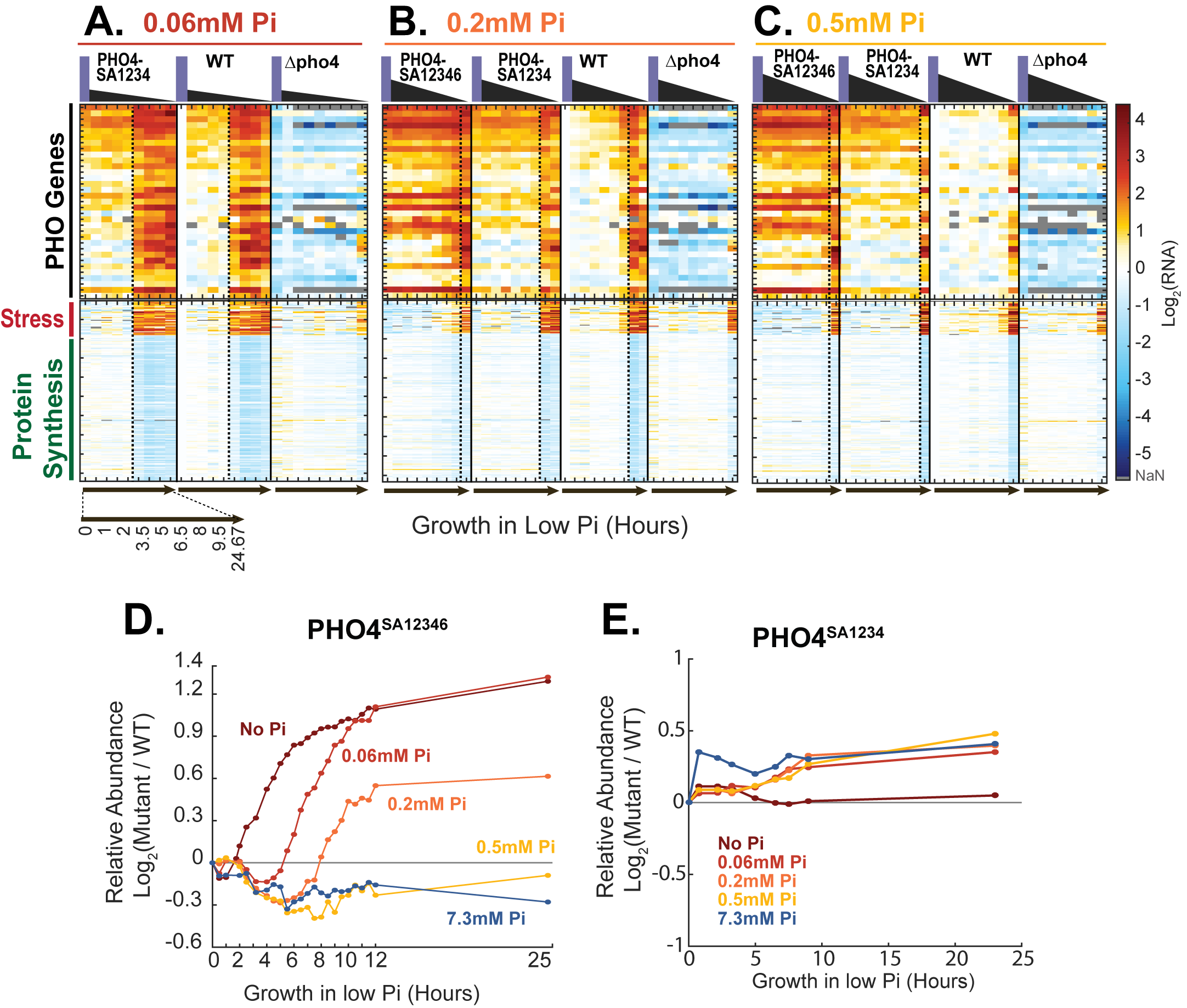

Supplement: S3 Fig — (A-C) Gene expression profiles of cells expressing the constitutive PHO4 allele: Same as Fig 1C for the indicated strains. PHO4SA12346 is a strong activating allele, while PHO4SA1234 is a weak activating allele [19]. Δpho4 is a PHO regulon deficient mutant. The data acquired during growth in 0.06 mM Pi (A), 0.2 mM Pi (B), and 0.5 mM Pi (C) are from 1 replicate. See (Fig 3C) for PHO4SA12346 growth in 0.06 mM Pi. Notice, constitutive PHO4 allele strains ability to strongly (PHO4SA12346) or partially (PHO4SA1234) express the Pho4-target genes even in high Pi levels (prior to transfer into low Pi, t = 0), as shown previously [19]. This activation is maintained also during the initial hours of growth in low Pi. The absence of the regulon induction seen in Δpho4 (A-C) was also verified with a Venus reporter (an independent experiment that uses an independent construct) (see S2C Fig). (D-E) Constitutive expression of Pho4-depdent genes promotes growth in low phosphate: Same as Fig 3A for the indicated strains. Per each of the five low and high Pi media in (D-E) 1 replicate is shown. The data in (D) are supported by Fig 3G. The raw data for (D-E) are available in S4. The raw data for (A-C) are available in S2 Data. (TIF) [file pbio.2002039.s003.tif]

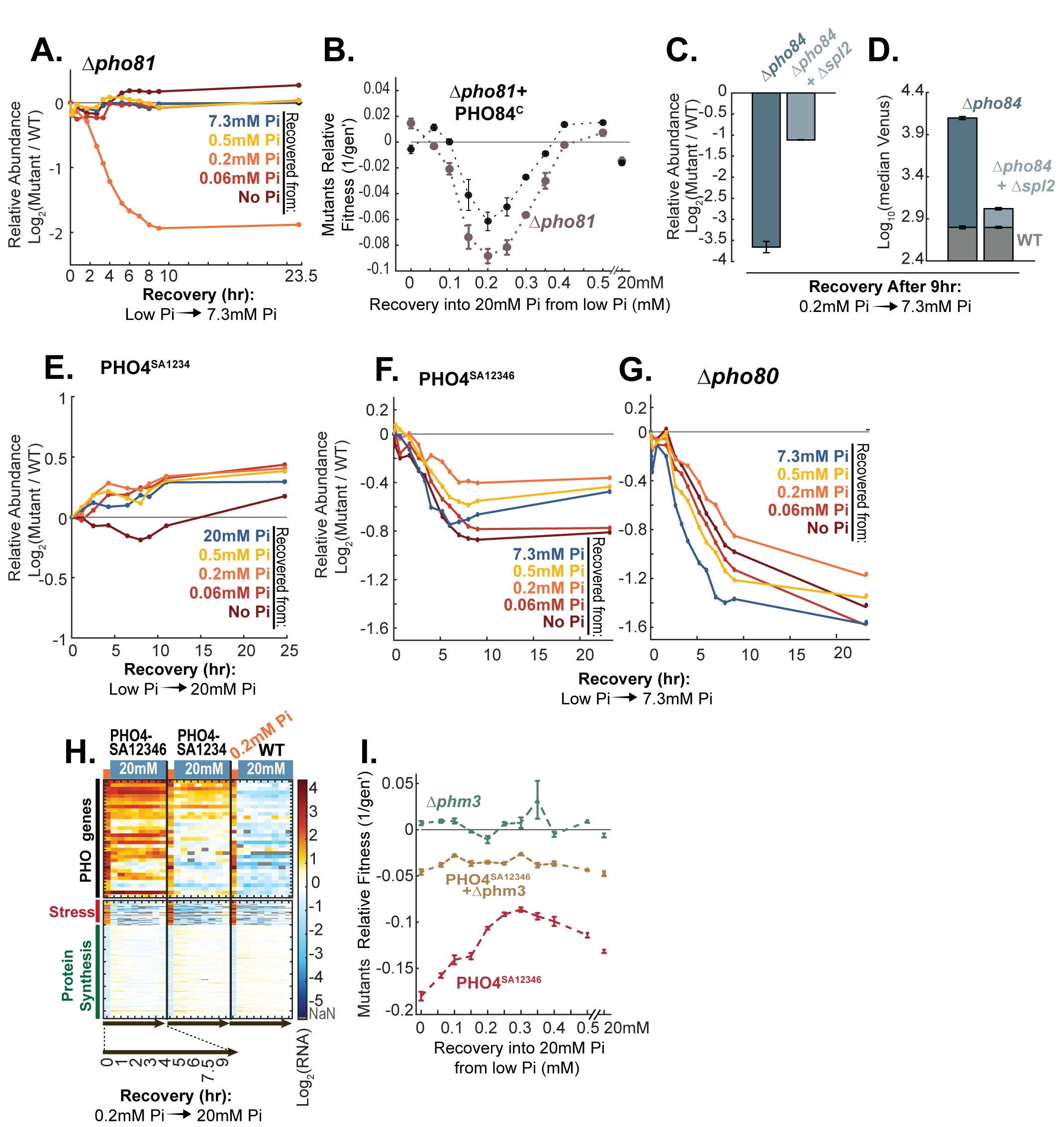

Supplement: S4 Fig — (A) The starvation program promotes recovery: Same as Fig 4C for Δpho81 cells. The data in (A) displays 1 replicate (per each of the 5 low and high Pi media) and is supported by the experiments shown in (B). (B) Constitutive expression of PHO84 partially compensates for the recovery phenotype of Δpho81 cells: same as Fig 4E for the indicated strains. The data are the mean and standard error of 3 replicates. (C-D) Both the low and high-affinity transporters are required for recovery from starvation: Same as Fig 4G and 4I for recovery into high Pi (7.3 mM Pi). The data in (C-D) are the mean and standard error of 2 replicates. (E-I) Strong constitutive activation of Pho4-target genes impedes recovery: (E-G) same as Fig 4C for the indicated strains. PHO4SA1234 and PHO4SA12346 are the weak and strong activating PHO4 alleles, respectively [19]. The data in (E-G) displays 1 replicate (per each of the five low and high Pi media). Data in (F) is supported by the experiments shown in (I). (H) Expression profiles same as Fig 4B for the indicated strains during recovery. The data are from 1 replicate. (I) Δphm3 partially compensates for the recovery phenotype of constitutive activation of the PHO4SA12346 allele: same as Fig 4E for the indicated strains. The data in (I) are the mean and standard error of 3 replicates. The raw data are available in S5 Data. The raw data for (A, C-G) are available in S4 Data. For (H) the raw data are available in S2 Data. (TIF) [file pbio.2002039.s004.tif]

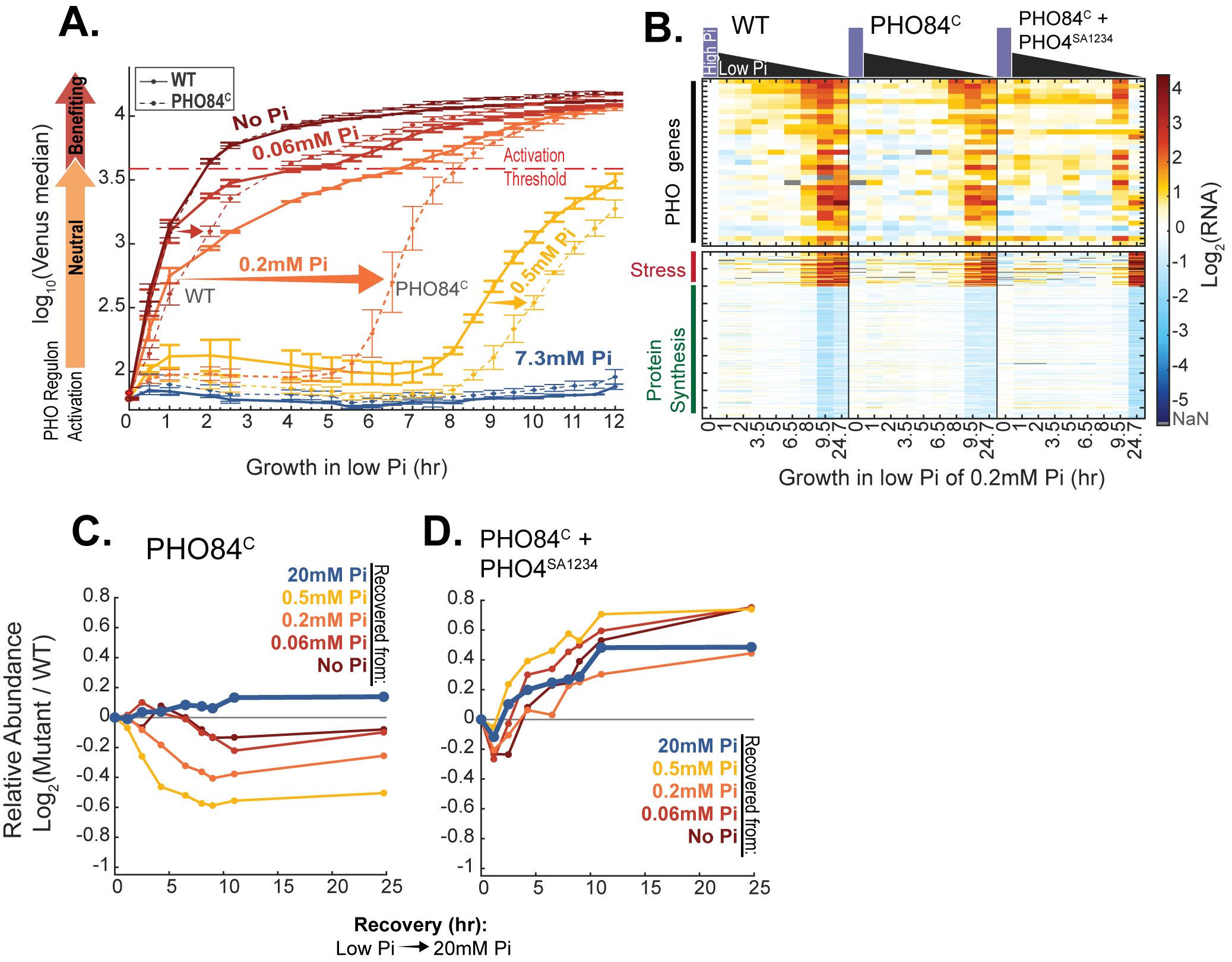

Supplement: S5 Fig — (A) The induction of Pho4-target genes reporter is delayed in PHO84C cells: wild-type and PHO84C cells expressing PHO84p-Venus reporter were mixed and transferred to media containing different levels of phosphate, same as S2C Fig. Cells were followed for twelve hours. Shown is the reporter expression in the 2 strains as a function of incubation time. The data in (A) are the mean and standard error of 3 replicates. (B) Induction of early wave of Pho4-target genes is delayed in PHO84C cells: Same as Fig 1C for the indicated strains. The data in (B) are from 1 replicate and is supported by Venus reporter experiments shown in (A). (C-D) Weak constitutive activation of Pho4-target genes rescue recovery of PHO84C cells: same as Fig 4C for the indicated strains. PHO4SA1234 is the weak PHO4 allele. Cells were recovered into high Pi of 20 mM Pi after 23 hours in the indicated low Pi levels. The data in (C-D) displays 1 replicate (per each of the five low and high Pi media), and supports previous report [22]. The raw data for (A, C-D) are available in S4 Data. The raw data for (B) are available in S2 Data. (TIF) [file pbio.2002039.s005.tif]

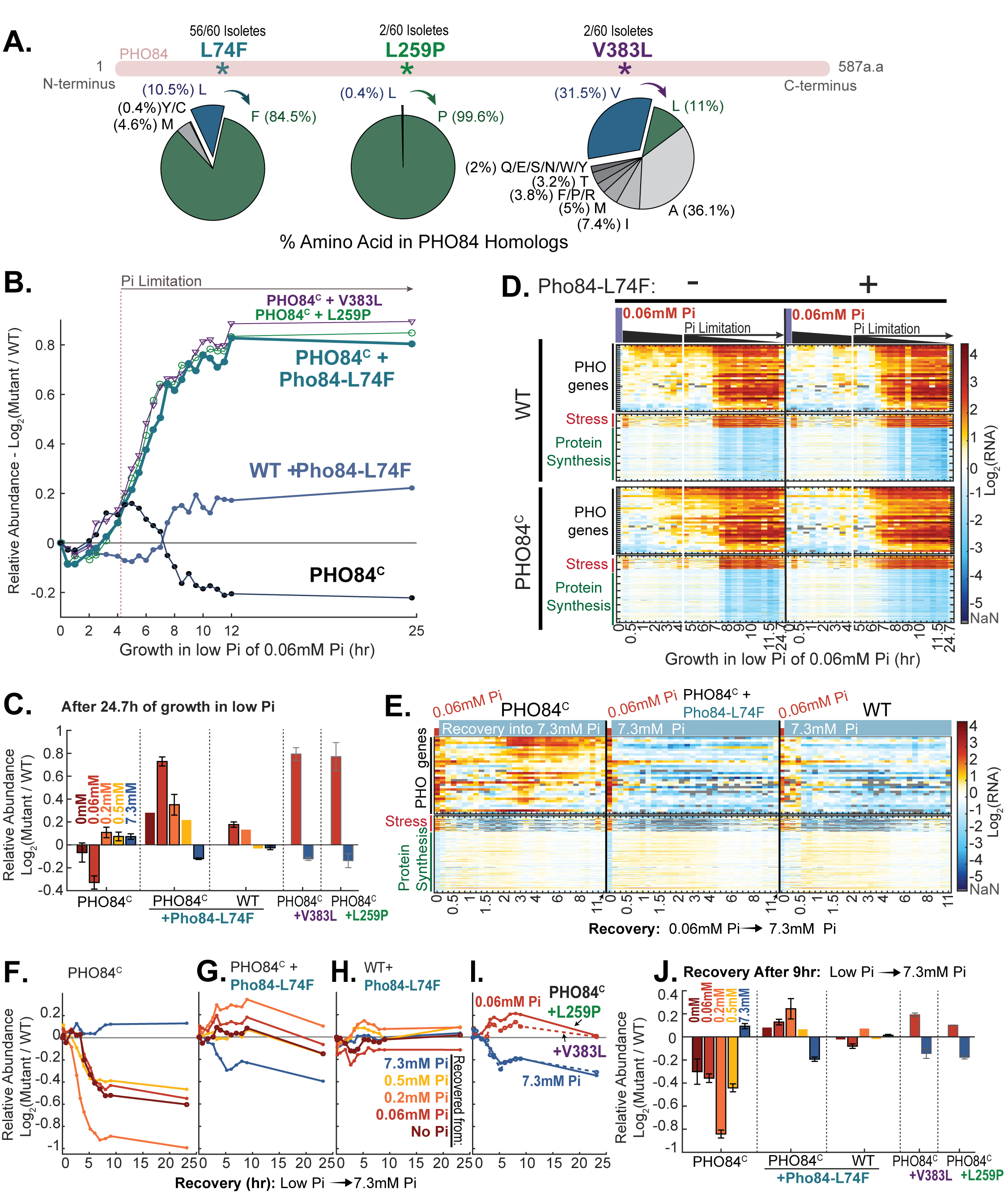

Supplement: S6 Fig — (A) Lab evolution selected for mutations that appear in PHO84 homologs: shown are the 3 amino-acid substitutions (Pho84-L74F, Pho84-V383L, and Pho84-L259P) identified in 60 evolved isolates, as described in Fig 6B and their presence in PHO84 homologs from different species (Materials and methods).The natural polymorphism of leucine to proline (L259P), is highly conserved and was previously suggested to be adaptive during phosphate starvation [48,49] (B-C) The selected PHO84 mutation promotes growth in low Pi: (B) Same as Fig 2B following transfer to a medium containing 0.06 mM Pi for the indicated strains. The PHO84 mutations (L74F (ttG/ttC), V383L, and L259P) identified in the selected stains (see Fig 6B) were introduced into wild-type and PHO84C cells instead of the wild-type allele (in PHO84C cells both the constitutive promoter and the evolved mutations were introduced into PHO84 endogenous site). The data in (B) are from 1 replicate and is supported by the experiments shown in (C). In (C) shown is the (log2) relative abundance after 24.7 hours of growth in the indicated low and high Pi levels. The data in (C) are the mean and standard error of 3, 2, or 1 replicates (see bar edge color: black, grey, or none, respectively). (D) The Pho84-L74F alters the temporal induction of Pho4-target genes: transcription profiles, as in Fig 1C, following transfer to a medium containing 0.06 mM Pi for the indicated strains. The data in (D) are from 1 replicate and is supported by the experiments with a Venus reporter (see S5A Fig and D4 Data). (E-J) Selected PHO84 mutation rescues the recovery phenotype: Same as Fig 6C–6E for the indicated mutations and starvation conditions. The data in (E) are from 1 replicate and is supported by the experiments with a Venus reporter, see D4 Data. The data in (F-I) are from 1 replicate and is supported by the experiments shown in (J). the data in (C) are the mean and standard error of 3, 2, or 1 replicates (see bar edge color: black, grey, or [file pbio.2002039.s006.tif]
